# Changes Over Time in HIV Prevalence and Sexual Behaviour Among Young Female Sex-Workers in 14 Sites in Zimbabwe, 2013–2016

**DOI:** 10.1007/s10461-019-02410-1

**Published:** 2019-02-20

**Authors:** Sungai T. Chabata, Bernadette Hensen, Tarisai Chiyaka, Phillis Mushati, Sibongile Mtetwa, Dagmar Hanisch, Sue Napierala, Joanna Busza, Sian Floyd, Elizabeth Fearon, Isolde Birdthistle, James R. Hargreaves, Frances M. Cowan

**Affiliations:** 1 Centre for Sexual Health, HIV/AIDS Research (CeSHHAR) Zimbabwe, 09 Monmouth Road, Avondale West, Harare, Zimbabwe; 2 Department of Clinical Research, Faculty of Infectious and Tropical Diseases, London School of Hygiene and Tropical Medicine, London, UK; 3 United Nations Population Fund, Harare, Zimbabwe; 4 Women’s Global Health Imperative, RTI International, San Francisco, CA USA; 5 Department of Population Health, Faculty of Epidemiology and Population Health, London School of Hygiene and Tropical Medicine, London, UK; 6 Department of Infectious Disease Epidemiology, Faculty of Epidemiology and Population Health, London School of Hygiene and Tropical Medicine, London, UK; 7 Department of Social and Environmental Health Research, Faculty of Public Health and Policy, London School of Hygiene and Tropical Medicine, London, UK; 8 Department of International Public Health, Liverpool School of Tropical Medicine, Liverpool, UK

**Keywords:** HIV prevention, Young female sex-worker, Adolescent girls and young women, Young women who sell sex, Zimbabwe

## Abstract

Young female sex-workers (FSW) aged 18–24 are at high risk of HIV due to high numbers of sexual partners, difficulty negotiating condom use, increased risk of gender-based violence, and limited access to services. Here we describe changes in sexual behaviours among young FSW across Zimbabwe between 2013 and 2016, and risk factors for prevalent HIV in 2013 and 2016. FSW ≥ 18 years were recruited using respondent-driven sampling in 14 sites across Zimbabwe in 2013 and 2016 as part of the SAPPH-IRe trial. We collected data on socio-demographics and sexual behaviour and offered HIV testing. Statistical analyses were RDS-II weighted. Characteristics of young FSW aged 18–24 were described, stratified by age. Logistic regression was used to assess difference in sexual behaviours by reported HIV status between 2013 and 2016, and to explore associations with prevalent HIV in 2013 and 2016. 656 young FSW were recruited in 2013 and 503 in 2016. Characteristics of young FSW were similar across both surveys. HIV prevalence was similar at both time points (35% vs 36%) and rose steeply with age. Compared to young FSW in 2013, reported condom-less sex with a steady partner and condom-less sex with clients was higher in 2016 among women self-reporting HIV negative status (OR = 6.41; 95%CI: 3.40-12.09; P<0.001) and (OR = 1.69; 95%CI: 1.14-2.51, P = 0.008), respectively, but not among young FSW self-reporting HIV positive status (OR = 2.35; 95%CI: 0.57-9.76; P = 0.236) and (OR = 1.87; 95%CI: 0.74-4.74; P = 0.186). After adjusting for age in 2016, young FSW who had ever been married had increased odds of testing HIV positive (OR = 1.88; 95% CI 1.04–3.39; P = 0.036) compared with those who had never married. Young FSW who completed secondary education or higher were less likely to test HIV positive (OR = 0.41; 95% CI 0.20–0.83; P = 0.012) compared with those with primary education or less. Young FSW remain at very high risk of HIV. Strategies to identify young FSW when they first start selling and refer them into services that address their economic, social and sexual vulnerabilities are critical.

**Electronic supplementary material:**

The online version of this article (10.1007/s10461-019-02410-1) contains supplementary material, which is available to authorized users.

## Introduction

In sub-Saharan Africa, the burden of HIV is higher among women than men, and adolescent girls and young women (AGYW) bear the greatest burden of new infections [1]. In 2016, AGYW (aged 15–24 years) accounted for more than 60% of new infections among those aged 15–24 [1]. Young female sex-workers (FSW) are at particularly heightened HIV risk. In addition to the physiological, emotional and social vulnerabilities faced by AGYW as they transition into adulthood, young FSW face added challenges related to stigma, discrimination and criminalisation [2, 3], and reduced ability to negotiate condom use with sexual partners [4]. This results in a ‘perfect storm’ of synergistic vulnerabilities that increase their susceptibility to HIV, sexually transmitted infections (STIs) and unintended pregnancy, as well as violence, poor mental health and substance use. As a consequence, young FSW are a particularly important group for comprehensive prevention interventions [5, 6].

Despite their increased risk of HIV, STIs and unplanned pregnancies, young FSW are poorly engaged with sexual health and HIV prevention and care programmes, in part because of fear of stigma and discrimination from healthcare providers, older FSW, families and friends, and the possible legal repercussions of visiting healthcare services and disclosing their engagement in sex work [5, 7, 8].

Data on socio-economic characteristics and HIV risk behaviours among young FSW remain sparse in Africa, including in Zimbabwe, due to their lack of engagement in health services [7] and difficulty in reaching them for research. The SAPPH-IRe trial (Sisters Antiretroviral Programme for Prevention of HIV, an Integrated Response PACTR201312000722390) [9, 10] tested a community intervention to improve FSW engagement with prevention and care services in Zimbabwe. Using data from this trial we explored the characteristics and sexual behaviours of young FSW aged 18–24 at two time-points prior to the roll-out of the US Government’s DREAMS (Determined, Resilient, Empowered, AIDS-free, Mentored and Safe) Partnership in Zimbabwe. Through the delivery of a combined package of HIV prevention interventions, DREAMS aims to reduce the risk of HIV among the most vulnerable AGYW, including young women who sell sex (YWSS), in ten sub-Saharan African countries [11]. We investigated trends in sexual risk behaviours over this time period to better interpret any future changes in behaviour following the introduction of DREAMS in Zimbabwe. We investigated whether these behaviours were associated with prevalent HIV in 2013 and 2016 [9]. Our hypothesis was that HIV prevalence would be higher among women reporting more years of sex work, and sexual risk behaviours would become less risky over time and differ by age. We aimed to identify characteristics and behaviours that put young FSW at high risk of HIV, which might be necessary for DREAMS and similar programmes to target among AGYW going forward.

## Methods

### Study Setting

The SAPPH-IRe trial was conducted in 14 communities in Zimbabwe where a national sex worker HIV prevention programme, ‘Sisters with a Voice’, had been running. The trial is described in detail elsewhere [10]. Briefly, the trial aimed to estimate the effect of a combination intervention on the proportion of all FSW who had a viral load ≥ 1000 copies/mL (reflecting whether an individual is infectious) [9]. The trial was completed in 2016, before the introduction of the DREAMS Partnership, allowing us to describe the risk environment among young FSW prior to the roll-out of DREAMS. The trial sites were purposively selected to reflect a range of settings [9]. Women were eligible to participate in the trial baseline and endline surveys if they were aged 18 years or older, had exchanged sex for money or gifts in the preceding 30 days, and had lived at the site for at least the previous 6 months.

### Data Sources

In this study, we used data from the SAPPH-IRe trial baseline and endline cross-sectional surveys. The baseline survey was conducted between 13th November and 20th December 2013 (2013 survey) and the endline survey between 11th April and 6th May 2016 (2016 survey). The surveys used respondent-driven sampling (RDS) to recruit two independent samples of FSW [9, 10, 12]. In these settings, sex is not commonly sold within brothels or other sex work specific venues, making RDS the most appropriate sampling method. As described elsewhere, prior to the RDS survey, geographic and social mapping was conducted over 2 to 3 days in each site [9]. This included informal discussions with trained peer educators, healthcare staff, and community informants. The mapping defined the geographic and social typology of sex work, determined if sex-workers at that site were well networked, and allowed for identification of FSW representative of these typologies (termed ‘seeds’) to initiate the RDS recruitment chains. In each location, between 6 and 8 seeds were invited to attend the survey site to complete a questionnaire and had a dried blood spot (DBS) taken for HIV antibody testing and, if positive, HIV viral load testing. All participants were also offered free HIV testing at the survey sites [10]. Seeds were then given two recruitment coupons to pass on to FSW peers. The women receiving a coupon (‘recruits’) who attended for interview were also given two coupons to give out to FSW in that location. We used an in-house coupon manager software to track coupons and all coupons were verified and redeemed only once. In all 14 sites, five iterations (‘waves’) of this process were performed for each survey. The sample size for each site was approximately 200 women, based on trial considerations [9]. The estimated population of FSW across these 14 sites ranged from 371 to 1409 therefore a sample of 200 women per site accounted for 14–54% of the target population across sites.

### Key Outcomes and Measures

Data were restricted to women recruited who were aged 18–24. The primary outcome of interest was HIV prevalence among young FSW. Sexual risk behaviours explored included those related to sex work characteristics (e.g. duration of selling sex, number of clients per week), condom use at last sex, and condom-less sex with steady partners and/or clients. Condom-less sex was defined as not having used a condom with a sexual partner at least once in the last month. The questions used to assess condom-less sex were: “In the past month, how often did you use condoms with your steady partner?”; “In the past month, has there been an occasion when you did not use condoms with your steady partner?”; “In the past month, how often did you use condoms with your clients?” and “Think again about all your clients in the last month, have there been any times when you did not use condoms?” We measured common mental disorders using the validated Shona Symptom Questionnaire (SSQ-8) [13], which asks eight questions about mental health symptoms experienced in the previous 1 week. The SSQ-8 is a screening tool for common mental disorders, including depression and anxiety [13–15]. A score of six or more out of eight indicates risk of common mental disorders. Insufficient quantity of food was defined as reporting spending at least 1 day without eating in the past month because there was no food in the household.

## Laboratory Methods

Blood samples were air-dried on filter papers and stored at room temperature until transported bi-weekly to the Flowcytometry Laboratory in Harare. If HIV antibodies were detected using AniLabsytems EIA kit (AniLabsystems Ltd, OyToilette 3, FIN-01720, Finland) then the sample was retested for HIV viral load using NucliSENS EasyQ HIV-1 v2.0, both to confirm HIV positive status and to quantify the viral load. For samples with a positive HIV antibody test using Anilab EIA, but an undetectable viral load, a second confirmatory ELISA was performed (Enzygnost Anti-HIV 1/2 Plus ELISA (Germany).

### Data Analyses

We follow the STROBE-RDS guidelines in reporting our study by first describing the samples recruited at each survey site [16]. RDS makes a number of assumptions including that purposively selected seeds do not bias the final estimates and that the target population is networked. We generated recruitment trees to judge onward recruitment by FSW in both surveys. We assessed whether final estimates of key outcomes converged over the five waves of recruitment and whether the social networks of FSW appeared to have been disconnected (bottlenecks) using combined convergence and bottleneck plots in each site. We also assessed recruitment homophily with respect to age and prevalent HIV to understand if recruiters were more likely to recruit women of their own age and if women who tested HIV positive were more likely to recruit women who tested HIV positive, respectively.

All analyses were RDS-II weighted, with women’s responses weighted by the inverse of the reported number of sex workers they knew i.e. the number of other women that she could have recruited to the survey [17]. The rationale for RDS-II weighting has been described in detail elsewhere [12, 18]. We pooled data from across the fourteen sites and both time points and normalised the RDS-II weights by site, dropping seeds and including a fixed term for site in all regression analysis. In order to minimise the risk of duplicate enrolment in each survey, we constructed a check identifier for each participant consisting of the first letter of her first name, the last three letters of her surname and her date of birth. Comparing check identifiers and questionnaire responses between 2013 and 2016 surveys suggested that fifteen young FSW (2% of those enrolled in 2013; 3% of those enrolled in 2016) were likely to have participated in both surveys. HIV prevalence and sexual behaviour estimates did not change after excluding the fifteen individuals, therefore, in both years, all participants were retained in the analysis. Socio-demographic characteristics and sexual risk behaviours were analysed descriptively among young FSW aged 18–19 and aged 20–24. Where there were a small number of women within categories of a categorical variable, for example duration in sex work, variables were collapsed into fewer categories to overcome issues of sparse data.

Weighted logistic regression models, with sexual risk behaviour as outcome and survey year as exposure were used to assess evidence for changes in sexual risk behaviours between the 2013 and 2016 surveys, separately for young FSW who self-reported HIV negative status and those who self-reported HIV positive status. This was done separately by self-reported HIV status, to understand if adoption of safer sexual behaviours differed between women who reported knowing their HIV negative or positive status.

We intentified the socio-economic factors and sexual risk behaviours associated with prevalent HIV (rapid HIV test results) at each survey, by first using univariable logistic regression. Factors associated with prevalent HIV at P ≤ 0.10 in univariable analysis were included into multivariable regression models, firstly adjusted for age at survey, and secondly adjusted for all factors that were associated with prevalent HIV in the univariable analysis with some conceptual framework for how these variables might be related, to understand factors independently associated with prevalent HIV. Age at time of the survey was included categorically. 2013 data will be presented in the Online Appendix and 2016 data in the main paper.

## Results

A total of 2722 FSW were recruited in 2013, of whom 24% (n = 656) were young FSW. In 2016, 2883 FSW were recruited, of whom 17% (n = 503) were young FSW. Extensive RDS diagnostics have been reported elsewhere [10, 19] and the findings apply to these data. Here we report RDS diagnostics of prevalent HIV and sexual risk behaviours.

In the 2013 survey, 90 FSW were seeds, 2804 FSW were examined for eligibility, and 2722 (97%) were eligible for the study. Overall, 3449 coupons were distributed of which 2714 (79%) were returned. In the 2016 survey, 92 FSW were seeds, 3025 FSW were examined for eligibility, and 2883 (95%) were eligible for the study. Overall, 3977 coupons were distributed of which 2933 (74%) were returned.

**Fig. 1:**
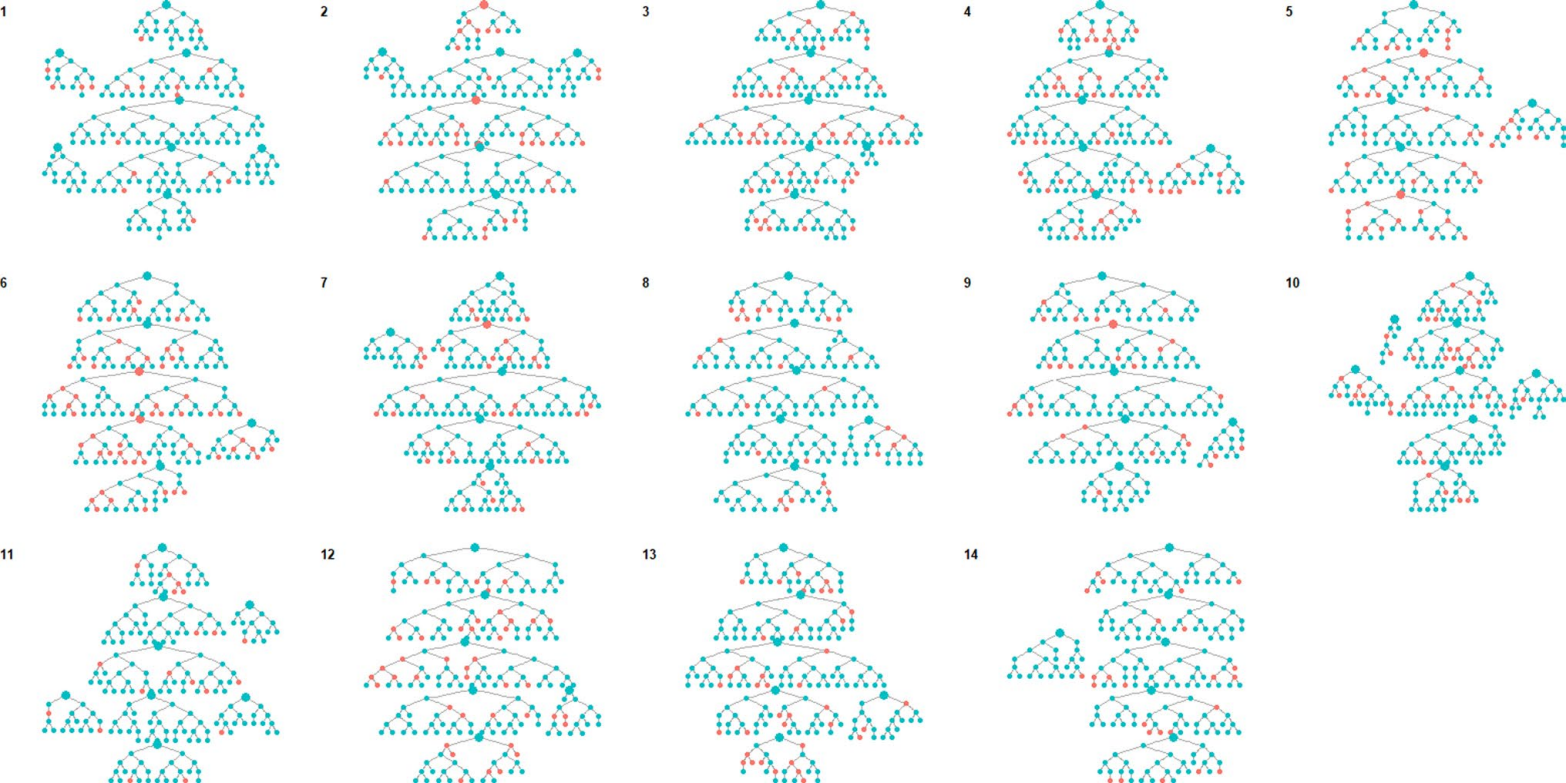
Recruitment tree diagrams. Participants are depicted by circles with their recruits shown as the connected circles below them. The larger circles denote seeds. Red circles represent women aged 18–24 and blue circles represent women aged ≥ 25 (Colour figure online)

**Fig. 2:**
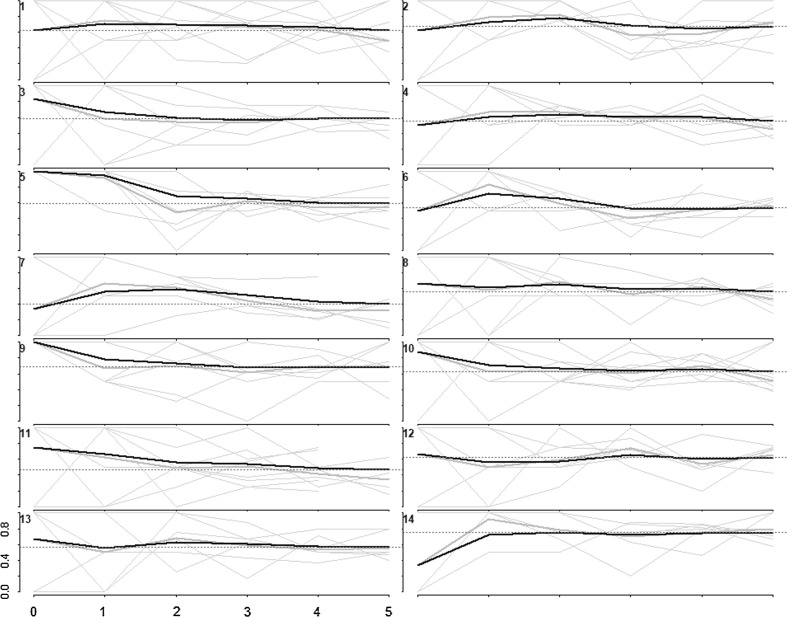
Convergence of the proportion of HIV positive FSW. The heavy black lines indicate the cumulative RDS-II weighted estimate overall for each site, while the grey lines are unweighted proportions for each seed, by sample wave (Colour figure online)

In the 2013 (Online Appendix 1) and 2016 surveys (Fig. 1), seeds were productive in recruiting FSW. In the 2013 survey, the number of recruits by seed ranged from 5 to 59 and in 2016 survey from 8 to 59 across sites. We judged that convergence appeared to have been achieved by the final sample size for HIV prevalence and that social networks of FSW in the respective sites were well connected (Fig. 2). This was true for sexual risk behaviours in 2013 and 2016 surveys (Online Appendix 1). Recruitment homophily on age and HIV prevalence was low in 2013 and 2016 surveys (Online Appendix 1).

The socio-demographic and behavioural characteristics of young FSW were similar across both surveys (Table 1). Two thirds of young FSW had some secondary education or higher. In 2013, 63% of young FSW aged 18–19 had never married versus 49% in 2016. Among young FSW aged 20–24, a similar proportion had never married in 2013 and 2016 (32% vs 31%). Overall, around a quarter of young FSW reported initiating sex work before the age of 18 in both surveys. In 2013, 27% of young FSW aged 18–19 reported ≥ 10 clients in the last week compared to 35% in 2016. Among young FSW aged 20–24, a similar proportion reported ≥10 clients in the last week in 2013 and 2016 (27% vs 30%). A higher proportion of young FSW aged 18–19 were aware of their of HIV positive status in 2016 compared to 2013 (54% vs 45%) and this was true for young FSW aged 20–24.

**Table 1:** Socio-demographic and sexual behavioural characteristics of young FSW by age, 2013 (N = 656) and 2016 (N = 503) (RDS-II weighted)

	2013 (N = 656)				2016 (N = 503)			
	18–19 years		20–24 years		18–19 years		20–24 years	
	N = 109		N = 547		N = 51		N = 452	
	n	(%)	n	(%)	N	(%)	n	(%)
Marital status								
Married/ living together as if married	0	0.0	3	0.5	1	0.8	3	1.6
Divorced/ separated	45	37.5	343	64.9	19	49.5	294	66.4
Widowed	0	0.0	19	2.8	1	0.8	8	1.0
Never been married	64	62.6	182	31.7	30	49.0	147	31.0
Highest level of education								
None	1	1.4	8	1.9	0	0.0	6	2.1
Primary level	38	34.8	106	21.3	13	30.4	87	22.1
Some secondary	52	49.0	229	44.3	28	55.8	178	39.2
Complete secondary or higher	17	14.8	203	32.5	10	13.9	181	36.6
Tribe								
Shona	85	68.3	427	74.0	41	81.3	334	71.9
Ndebele	18	24.3	47	11.4	7	10.5	48	11.2
Kalanga	0	0.0	11	1.4	0	0.0	1	0.1
Other	6	7.4	62	13.3	3	8.1	68	16.8
Religion								
Christian	56	57.2	267	49.0	32	62.1	263	53.2
Muslim	0	0.0	2	0.4	1	0.8	3	1.1
African traditional	0	0.0	1	0.2	0	0.0	1	0.2
Other	11	14.6	35	6.2	8	10.5	54	13.8
No religion	42	28.1	241	44.1	10	26.6	131	31.7
Number of children								
0	46	46.4	152	30.6	23	43.2	119	30.3
1–2	52	47.4	329	59.3	26	54.4	268	54.9
3 +	11	6.2	66	10.2	2	2.4	65	14.8
No food for one day in the past month								
No	70	63.0	325	60.0	36	67.4	308	67.7
Yes	39	37.0	222	40.0	15	32.6	143	32.3
Age at start of sex work								
≤ 15	19	16.2	26	5.6	9	18.6	21	5.9
16–17	56	51.8	63	9.6	32	69.7	68	12.6
18–24	34	32.0	458	84.7	10	11.7	362	81.5
Duration in sex work (years)								
0–2	82	79.7	300	60.5	36	71.4	204	46.7
3–4	22	15.6	165	26.0	14	28.2	161	34.5
≥ 5	5	4.7	82	13.5	1	0.4	86	18.8
Number of clients in the last week								
0–4	47	55.8	225	46.4	21	42.7	181	41.4
5–9	24	17.7	141	26.8	16	22.6	136	29.2
≥ 10	38	26.5	181	26.9	14	34.7	134	29.5
Relationship with other female sex-workers in one’s location								
Good	63	56.8	323	62.1	36	59.4	295	63.1
Neither good nor bad	31	28.6	174	28.7	12	31.8	132	33.0
Bad	8	6.5	32	5.8	1	0.3	15	2.2
No relationship	7	8.1	18	3.3	2	8.5	9	1.7
No. of female sex-workers who are close friends								
≤ 1	30	20.6	156	26.6	19	34.2	169	35.2
2–3	58	61.6	291	55.9	24	57.2	188	44.6
≥ 4	21	17.8	100	17.5	8	8.6	94	20.2
Alcohol consumption in the past 12 months								
Never	42	47.4	184	36.1	19	30.7	131	31.4
Once a month or less	8	5.9	59	12.5	3	7.4	35	6.4
2–4 times per month	14	9.2	77	13.7	4	2.3	51	11.8
2–3 times per week	23	20.4	79	13.1	13	27.1	116	27.2
4 or more times per week	22	16.9	148	24.6	12	32.4	118	23.2
Symptoms of common mental disorder								
No	58	53.3	268	51.4	41	84.8	311	68.2
Yes	51	46.7	276	48.6	10	15.2	138	31.8
Experience of physical violence from steady partner								
No	63	62.7	305	59.5	27	50.4	242	58.3
Yes	46	37.3	242	40.5	24	49.6	209	41.7
Experience of physical violence from client								
No	82	77.1	397	76.2	29	52.6	327	75.9
Yes	27	22.9	150	23.8	22	47.4	124	24.1
Ever forced to have sexual intercourse								
No	100	90.4	528	96.4	46	90.7	412	89.9
Yes	9	9.6	19	3.6	5	9.3	39	10.1
Self-reported HIV status								
Negative	80	85.0	403	82.8	43	86.8	354	74.7
Positive	12	15.0	100	17.2	5	13.2	88	25.3
Rapid HIV test result								
Negative	80	73.3	339	63.0	39	79.3	290	62.0
Positive	28	26.7	208	37.0	12	20.7	161	38.0
Knowledge of HIV positive status^a^								
No	18	55.4	116	61.0	9	45.7	77	38.1
Yes	10	44.6	92	39.0	3	54.3	84	61.9

^a^Proportion reported HIV positive among those who tested HIV positive during the survey

**Fig. 3:**
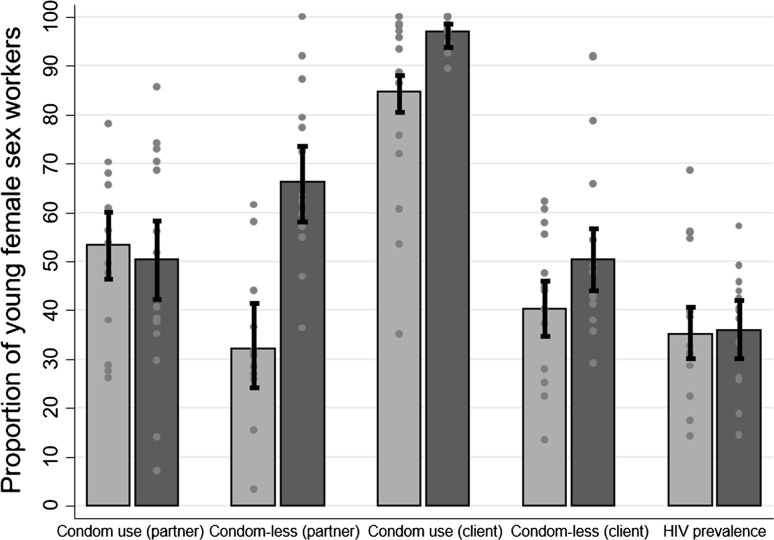
Condom use at last sex and condom-less sex with steady partner, condom use at last sex and condom-less sex with client, and HIV prevalence overall among young female sex-workers (aged 18–24) and by site (grey spots  =  sites; light blue bars = 2013 survey; dark blue bars = 2016 survey) (Colour figure online)

Between 2013 and 2016, reported sexual behaviours changed most among women who self-reported their HIV negative status. In 2016, there was evidence that a higher proportion of young FSW who self-reported their HIV negative status were in sex work for ≥ 3 years relative to 2013 (48% vs 35%, respectively; OR = 1.68; 95% CI 1.13–2.49; P = 0.010) (Table 2). In 2016, young FSW reporting HIV negative status were more likely to report having a steady partner compared to 2013 (OR = 1.63; 95% CI 1.07–2.47; P = 0.022). Compared to young FSW participating in the 2013 survey, condom-less sex with a steady partner in the past month was higher in 2016 among women self-reporting HIV negative status (31% vs 70%, respectively; OR = 6.41; 95% CI 3.40–12.09; P<0.001) but not among women self-reporting HIV positive status (OR = 2.35; 95% CI 0.57–9.76; P = 0.236), and condom-less sex with clients in the past month evidently increased among women who reported being HIV negative (OR = 1.69; 95% CI 1.14–2.51, P = 0.008) but there was no evidence of an increase among those who reported knowing their HIV positive status (OR = 1.87; 95% CI 0.74–4.74; P = 0.186). Figure 3 shows the overall changes in condom variables and HIV prevalence between 2013 and 2016, with site specific values denoted by dots to show the variability of proportions and prevalence across sites. There was evidence that ever being forced to have sex was higher in 2016 relative to 2013 among women who reported being HIV negative (11.1% vs 4.5%, respectively; OR = 2.63; 95% CI 1.03–6.75; P = 0.044).

**Table 2:** Comparison of sexual risk behaviour characteristics among young FSW, by self-reported HIV status, 2013 (N = 656) and 2016 (N = 503)

Characteristic	2013				2016				2016 vs 2013			
	18–24 years (N = 656)				18–24 years (N = 503)				18–24 years			
	HIV-negative		HIV-positive		HIV-negative		HIV-positive		HIV-negative		HIV-positive	
	N = 544		N = 112		N = 409		N = 93					
	n	% (95% CI)	n	% (95% CI)	n	% (95% CI)	n	% (95% CI)	OR (95% CI)^a^	P-value^b^	OR (95% CI)^a^	P-value^b^
Duration in sex work (years)												
0–2	321	64.6 (58.6–70.1)	61	60.3 (47.3–72.0)	201	52.5 (45.7–59.3)	39	40.7 (27.8–55.0)	–		–	
3+	223	35.4 (29.9–41.4)	51	39.7 (28.0–52.7)	208	47.5 (40.7–54.3)	54	59.3 (45.0–72.2)	1.68 (1.13–2.49)	0.010	1.88 (0.83–4.22)	0.126
Number of clients in the last week												
0–4	231	49.5 (43.4–55.5)	41	39.9 (27.9–53.4)	169	43.9 (37.3–50.8)	33	33.6 (21.8–47.9)	–		–	
5+	313	50.5 (44.5–56.6)	71	60.1 (46.6–72.1)	240	56.1 (49.2–62.7)	60	66.4 (52.1–78.2)	1.27 (0.87–1.86)	0.213	1.19 (0.55–2.60)	0.656
Has a steady partner												
No	194	36.5 (30.7–42.6)	43	35.6 (24.0–49.3)	123	27.0 (21.8–33.0)	32	42.3 (28.7–57.1)	–		–	
Yes	350	63.5 (57.4–69.3)	69	64.4 (50.7–76.0)	286	73.0 (67.0–78.2)	61	57.7 (42.9–71.3)	1.63 (1.07–2.47)	0.022	0.89 (0.39–2.06)	0.791
Condom use at last sex with steady partner												
No	154	47.0 (39.5–54.6)	30	44.8 (29.5–61.3)	141	51.7 (42.7–60.6)	24	41.3 (24.4–60.6)	–		–	
Yes	178	53.0 (45.4–60.5)	33	55.2 (38.7–70.5)	114	48.3 (39.4–57.3)	35	58.7 (39.4–75.6)	0.88 (0.54–1.44)	0.614	1.45 (0.44–4.73)	0.535
Condom–less sex with steady partner in the past month												
No	124	68.6 (58.3–77.3)	21	63.3 (39.9–81.7)	68	29.7 (22.0–38.7)	26	48.9 (31.2–67.0)	–		–	
Yes	54	31.4 (22.7–41.7)	12	36.7 (18.3–60.1)	187	70.3 (61.3–78.0)	33	51.1 (33.0–68.8)	6.41 (3.40–12.09)	<0.001	2.35 (0.57–9.76)	0.236
Condom use at last sex with client												
No	94	15.2 (11.5–19.9)	18	15.3 (7.9–27.5)	10	2.8 (1.2–6.3)	2	3.5 (0.7–15.4)	–		–	
Yes	450	84.8 (80.1–88.5)	94	84.7 (72.5–92.1)	399	97.2 (93.7–98.8)	90	96.5 (84.6–99.3)	8.15 (2.56–25.93)	<0.001	9.34 (1.84–47.33)	0.007
Condom-less sex with client in the past month												
No	300	60.8 (54.5–66.7)	51	53.7 (40.1–66.9)	200	48.4 (41.4–55.5)	44	53.8 (38.9–68.0)	–		–	
Yes	199	39.2 (33.3–45.5)	49	46.3 (33.1–59.9)	185	51.6 (44.5–58.6)	42	46.2 (32.0–61.1)	1.69 (1.14–2.51)	0.008	1.87 (0.74–4.74)	0.186
Failed to use a condom with client as a result of own drinking during the past 12 months												
No	254	79.0 (73.0–84.0)	50	69.8 (54.5–81.6)	234	83.5 (76.4–88.7)	57	87.2 (73.0–94.5)	–		–	
Yes	101	21.0 (16.0–27.0)	21	30.2 (18.4–45.5)	49	16.5 (11.3–23.6)	11	12.8 (5.5–27.0)	0.79 (0.45–1.37)	0.400	0.37 (0.11–1.30)	0.122
Failed to use a condom with client as a result of client’s drinking during the past 12 months												
No	456	86.4 (81.8–89.9)	91	76.4 (61.6–86.6)	383	92.1 (87.0–95.3)	85	90.3 (78.0–96.1)	–		–	
Yes	86	13.6 (10.1–18.2)	21	23.6 (13.4–38.4)	26	7.9 (4.7–13.0)	8	9.7 (3.9–22.0)	0.57 (0.29–1.10)	0.092	0.26 (0.07–1.02)	0.053
Ever forced to have sexual intercourse												
No	522	95.5 (91.8–97.5)	106	94.5 (86.7–97.8)	371	88.9 (83.9–92.4)	87	93.7 (84.2–97.7)	–		–	
Yes	22	4.5 (2.5–8.2)	6	5.5 (2.2–13.3)	38	11.1 (7.6–16.1)	6	6.3 (2.3–15.8)	2.63 (1.03–6.75)	0.044	1.68 (0.41–6.92)	0.472

^a^Including fixed term for site

^b^Wald P-value

In univariable analysis of factors associated with prevalent HIV in 2013 (Online Appendix 2), there was evidence that age and marital status were associated with prevalent HIV. However, there was no evidence of an association after adjusting for age at time of the survey, marital status and number of FSW who are close friends. In 2016, there was evidence that age, marital status, educational attainment and reported number of clients in the previous week were crudely associated with prevalent HIV (Table 3), with no evidence that HIV prevalence was higher among women selling sex for ≥ 5 years relative to women selling sex for 0–2 years (28.4% vs 38.1%; OR = 0.66; 95% CI 0.32–1.35; P = 0.302) and little evidence of an association with condom use variables, experience of violence or alcohol use.

**Table 3:** Factors associated with prevalent HIV among young FSW aged 18–24 in Zimbabwe, 2016 (N = 503)

Characteristic	N (%)	Number of young female sex-workers tested HIV-positive during the survey (n = 173) n (%)	Crude OR (95% CI)	P-value	Age adjusted OR (95% CI)	P-value	Adjusted^a^ OR (95% CI)	P-value
Age at time of survey				0.029				0.035
18–19	51 (12.5)	12 (20.7)	1				1	
20–24	451 (87.5)	161 (38.0)	3.06 (1.12–8.38)				3.09 (1.09–8.81)	
Marital status				0.013		0.036		0.060
Never married	176 (33.0)	41 (23.7)	1		1		1	
Ever married	326 (67.0)	132 (41.9)	2.10 (1.17–3.77)		1.88 (1.04–3.39)		1.77 (0.98–3.21)	
Highest level of education				0.025		0.012		0.031
Primary school or less	105 (24.7)	47 (47.4)	1		1		1	
Some secondary school	206 (41.4)	76 (36.3)	0.66 (0.33–1.30)		0.68 (0.34–1.34)		0.69 (0.35–1.36)	
Complete secondary or higher	191 (33.9)	50 (26.9)	0.45 (0.22–0.90)		0.41 (0.20–0.83)		0.46 (0.22–0.94)	
Number of children				0.462				
0	141 (31.7)	54 (41.9)	1					
1–2	294 (55.0)	96 (32.1)	0.61 (0.34–1.10)					
≥ 3	67 (13.3)	23 (37.3)	0.89 (0.37–2.14)					
Duration in sex work				0.302				
0–2	240 (49.8)	83 (38.1)	1					
3–4	175 (33.7)	56 (36.2)	0.92 (0.51–1.67)					
≥ 5	87 (16.5)	34 (28.4)	0.66 (0.32–1.35)					
Number of clients in the last week				0.068		0.043		0.090
0–4	202 (41.5)	56 (28.9)	1		1		1	
5–9	152 (28.3)	55 (37.7)	1.60 (0.84–3.05)		1.58 (0.82–3.04)		1.45 (0.74–2.85)	
≥ 10	148 (30.2)	62 (43.8)	1.79 (0.94–3.44)		1.88 (1.01–3.51)		1.70 (0.90–3.22)	
Condom use at last sex with steady partner				0.635				
No	165 (49.5)	54 (29.1)	1					
Yes	149 (50.5)	48 (32.5)	1.18 (0.59–2.38)					
Condom-less sex with steady partner in the past month				0.165				
No	94 (33.7)	33 (37.3)	1					
Yes	220 (66.3)	69 (27.6)	0.58 (0.27–1.25)					
Condom use at last sex with client				0.96				
No	12 (3.0)	3 (33.6)	1					
Yes	489 (97.0)	169 (35.6)	1.05 (0.15–6.03)					
Condom-less sex with client in the past month				0.277				
No	244 (49.6)	77 (36.6)	1					
Yes	227 (50.4)	80 (32.5)	0.73 (0.41–1.29)					
Experience of physical violence from steady partner				0.917				
No	269 (57.3)	87 (36.2)	1					
Yes	233 (42.7)	86 (35.4)	0.97 (0.58–1.63)					
Experience of physical violence from client				0.944				
No	356 (73.0)	114 (35.7)	1					
Yes	146 (27.0)	59 (36.4)	1.08 (0.54–1.77)					
Ever forced to have sexual intercourse				0.523				
No	458 (90.0)	154 (35.5)	1					
Yes	44 (10.0)	19 (39.4)	1.32 (0.57–3.06)					
Alcohol use in the past 12 months				0.699				
Never	150 (31.3)	46 (36.0)	1					
Once a month or less	38 (6.5)	12 (45.0)	1.61 (0.50–5.17)					
2–4 times per month	55 (10.6)	16 (20.2)	0.50 (0.20–1.24)					
2–3 times per week	129 (27.2)	47 (35.4)	1.09 (0.54–2.22)					
4 or more times per week	130 (24.4)	52 (40.4)	1.20 (0.58–2.48)					
No food for one day in the past month				0.376				
No	344 (67.6)	120 (37.4)	1					
Yes	158 (32.4)	53 (32.7)	0.78 (0.45–1.35)					
Relationship with other FSW in one’s location				0.499				
Good	331 (62.7)	110 (35.2)	1					
Neither good nor bad	144 (32.8)	53 (35.5)	1.12 (0.62–2.02)					
Bad or no relationship	27 (4.5)	10 (47.2)	1.94 (0.64–5.91)					
No. of FSW who are close friends				0.201				
≤ 1	188 (35.1)	70 (36.8)	1					
2–3	212 (46.1)	77 (40.8)	1.26 (0.68–2.31)					
≥ 4	102 (18.8)	26 (22.0)	0.47 (0.21–1.05)					
Symptoms of common mental disorder				0.795				
No	352 (70.3)	121 (36.3)	1					
Yes	148 (29.7)	51 (34.8)	0.93 (0.52–1.65)					

^a^Adjusted for age, marital status, highest level of education and number of clients in the last week

After adjusting for age, there was strong evidence that marital status, educational attainment and reported number of clients in the previous week were associated with prevalent HIV. Young FSW who had ever been married had a higher prevalence of HIV (adjOR = 1.88; 95% CI 1.04–3.39; P = 0.036) compared with those never married. Young FSW with complete secondary education or higher were less likely to test HIV positive compared to women with primary education or less (adjOR = 0.41; 95% CI 0.20–0.83; P = 0.012). Young FSW reporting ≥ 10 clients in the last week (adjOR = 1.88; 95% CI 1.01–3.51; P = 0.043) were more likely to be HIV positive compared to those reporting 0–4 clients in the last week.

Among women aged 20–24, HIV prevalence was 38.0% compared to 20.7% among women aged 18–19 and after adjusting for marital status, educational attainment and reported number of clients in the previous week, there was evidence that HIV prevalence differed by age (adjOR = 3.09; 95% CI 1.09–8.81; P = 0.035).

## Discussion

We used data from a completed trial to explore the characteristics and sexual behaviours of young FSW aged 18 to 24 at two time-points separated by 30 months, and investigated whether these factors were associated with prevalent HIV in 2013 and 2016. In both surveys, over one-third of the women tested HIV positive, confirming that young FSW in Zimbabwe are at consistently high risk of infection. A lower proportion were aware of their HIV positive status in 2013. As seen among other young women in southern Africa, HIV prevalence was higher amongst those young FSW who were divorced or separated [20]. Young FSW reported high numbers of clients in the previous week and high rates of condom-less sex in the past month with a steady partner or client. In 2016, we found that women who were ever married, had less education, and who reported a higher number of clients in the past week were more likely to test HIV-positive but found no evidence that duration of selling sex or reported condom use were associated with HIV.

In 2016, HIV prevalence among young FSW aged 20–24 was approximately four-times higher than the HIV prevalence of 10% among women aged 20–24 in the general population [21]. It was also 1.5 times higher than in young FSW aged 18–19, suggestive of high HIV incidence. This hypothesis is supported by a cohort analysis of the Sisters with a Voice programmatic data, which estimated an HIV incidence of 11% per year among women aged < 26 between 2009 and 2013 [22].

We hypothesised that HIV prevalence would be higher among women reporting more years of sex work but found no association; in 2016 HIV prevalence seemed to be decreasing with increased duration in sex work. Prevalent HIV was higher among women reporting more clients in the past week, highlighting the risk associated with “high activity” sex work. We also found an association with reporting ever being married, possibly because it is difficult for married women to use condoms with their husbands. Alternatively it is possible that women widowed by an HIV-positive husband or separated from their partner because of their HIV-positive status are more likely to transition into sex work, although we were not able to explore this in our survey data. A study among sex-workers attending a clinic in Kenya found that younger women were at higher risk of HIV infection, and that women in sex work for < 2 years at enrolment had 2.3-fold higher risk of HIV than women in sex work for > 2 years [23]. An earlier study in India among brothel-based sex-workers found that HIV prevalence was higher among sex-workers aged ≤20 compared to sex-workers aged 21 or older (12.5% vs 5.4%) [24]. A better understanding of the complex relationship between HIV risk and entry into sex work is required to effectively target HIV prevention and social protection services based on the needs of different groups of women.

Despite our hypothesis that sexual risk behaviours would become less risky between 2013 and 2016, analysis of sexual behaviours found that sexual risky behaviours actually became more risky within this period, particularly among women who reported to be HIV negative. This upward change in some risky behaviours was also seen in Thailand among men who have sex with men, an equally most-at-risk population [25]. Condom-less sex with steady partners increased in 2016 compared to 2013 but the magnitude of increase was higher among women who reported being HIV negative compared to those who reported their HIV status as positive. This might suggest that women aware of their HIV-positive status may adopt safer behaviours to reduce the risk of onward transmission to clients and partners, and points to the need for additional targeting of HIV negative young women. Or possibly that more women have steady partners who are aware of their HIV negative status.

Both reported condom use at last sex and condom-less sex in the previous month with clients increased between 2013 and 2016, which is contradictory. Reported condom use is subject to social desirability bias, so while it is possible these changes may reflect changes in actual condom use they could also reflect biases in reporting. Poor validity of self-reported sexual behaviours, particularly condom use, is well recognised [26, 27], with condom use at last sex considered at particular risk of over-reporting [28]. Similar to studies among FSW in India [29] and Cambodia [30], our analysis of factors associated with prevalent HIV found no association between condom use variables and HIV. HIV may have been acquired some time prior to the survey. Further, women may or may not be using condoms because they know their HIV status and/or their partners’ HIV status, or if they know that ART treatment is effective at preventing HIV transmission. Despite the complexities and limitations in understanding the relationship between recent condom use and prevalent HIV, reported condom-less sex in the past month was also high among HIV-negative young FSW. Although an important prevention tool, condom provision alone may be insufficient to protect young FSW from HIV, particularly without specific interventions to increase risk perception and improve condom negotiation skills. In recent years, trials have proven the effectiveness of oral pre-exposure prophylaxis (PrEP) in reducing HIV among women adherent to PrEP [31, 32]. The availability of PrEP provides an important alternate prevention tool for women, and an opportunity to reduce HIV incidence among this population of women.

After adjusting for age at the time of the survey in 2016, lower educational attainment was strongly associated with HIV. This finding is similar to trends observed in some, but not all, sub-Saharan African countries [33]. Studies among young women in the general population have shown that staying in school can reduce HIV risk in some settings [34, 35] and that educational attainment has a protective effect against HIV [36, 37]. Low educational attainment has also been shown to be associated with initiating selling sex, likely contributing to increased HIV risk [5, 38]. Our findings emphasise the need to identify female adolescents who are at risk of dropping out of school to either support them to remain in school or provide a safety net to reduce their future vulnerability to HIV [39]. Interventions such as the DREAMS partnership which address the structural drivers of HIV are likely important for preventing HIV in this population [11].

Our study is subject to limitations. The women participating in the survey were recruited through RDS. Due to the nature of RDS, we cannot empirically explore whether the sample of women participating in the survey is representative of FSW in the study sites. However, extensive diagnostic testing of the RDS data suggests little evidence of bias [10, 19], although, as we have reported elsewhere, there is some suggestion that women who engaged with the Sisters with a Voice programme were more likely to participate in the 2016 survey [19] which might explain social desirability bias in reporting of condom use at last sex because women are taught about the importance of condom use at programme visit. The surveys were cross-sectional and therefore we cannot explore the temporal relationship between risk factors and HIV. The check identifier we used to minimise the risk of duplicate enrolment in each survey was subject to women using different names and/or giving different date of birth. However, many participants aged 22 to 24 in 2013 (43%) would have aged out of eligibility by 2016, and additionally those aged 18 to 20 in 2016 (23%) would have been ineligible to take part in the 2013 survey. Despite these limitations, both surveys include a sizeable proportion of young FSW recruited using the same RDS procedures in 14 sites nationally. The findings provide critical insights into the vulnerability and HIV risk among this understudied population of women.

In conclusion, our findings confirm that young FSW in Zimbabwe are at a very high risk of HIV. The multicomponent DREAMS Partnership aims to reach YWSS, providing an important opportunity to offer these women a package of services to improve their health and well-being, tailored to address the findings from this research, such as condom negotiation, access to education, PrEP, and sexual and reproductive health services more generally. As part of DREAMS, PrEP is being offered to YWSS. To maximise the HIV prevention impact of PrEP, programmes will need to address structural factors such as costs and social factors such as stigma likely to hinder PrEP access and adherence among young FSW, which DREAMS partnership is designed to provide [11]. To support effective targeting of future strategies to reduce HIV risk, a better understanding of whether FSW’s higher HIV risk is due to a high risk when they first initiate sex work, or reflects higher risk prior to transitioning into sex work, is needed. Such information would help identify strategies to target young women when they are most at risk of HIV, and refer them into appropriate socioeconomic services and HIV-prevention and related health interventions.

## Electronic Supplementary Material

Below is the link to the electronic supplementary material.

Supplementary material 1 (PDF 1178 kb)
