# Supplementary material for: Changes Over Time in HIV Prevalence and Sexual Behaviour Among Young Female Sex-Workers in 14 Sites in Zimbabwe, 2013–2016
Source: AIDS Behav. 2019 Feb 20;23(6):1494–507. doi: 10.1007/s10461-019-02410-1 (PMC6535417; doi:10.1007/s10461-019-02410-1)
Supplement: Supplementary file 1 — Supplementary material 1 (PDF 1178 kb) [file 10461_2019_2410_MOESM1_ESM.pdf]

Appendix 1: Respondent Driven Sampling (RDS) diagnostics

Extensive RDS diagnostics for the SAPPH-IRe trial have been reported elsewhere (1, 2). Here we focus on the RDS diagnostics of our key outcomes for the two surveys; prevalent HIV and all the sexual risk behaviours we assessed for change over time (duration in sex work, number of clients in the last week, reporting of steady partner, condom use at last sex with steady partner, condom-less sex with steady partner, condom use at last sex with client, condom-less sex with client, ever forced to have sexual intercourse, failed to use a condom with client as a result of own drinking during the past 12 months, and failed to use a condom with client as a result of client's drinking during the past 12 months).

We generated recruitment trees to judge on-ward recruitment by female sex workers (FSW) aged 18-24 and 25 or more in the 14 sites. We assessed wether final estimates converged by the final sample size and whether the social networks of FSW appeared to have been disconnected (bottlenecks) using the combined convergence and bottleneck plots in each site. RDS makes an assumption that seed participants who are purposively sampled do not bias the final estimates and that the target population is networked. We also assessed recruitment homophily on age and prevalent HIV to understand if recruiters were more likely to recruit women of their age and if HIV positive recruiters were more likely to recruit HIV positive women respectively.

Seeds were productive in recruiting FSW into the surveys. We judged that overall, convergence appeared to have been achieved by the final sample size for prevalent HIV and all the sexual risk behaviour characteristics and that the social networks of FSW in the respective sites were well connected. However, there were very few cases, for example in site 5 in 2013 survey where the estimate of the proportion of FSW who reported to have failed to use a condom with client as a result of own drinking during the past 12 months might have continue to rise had the sample been increased. In this case, we might have

underestimated the estimate of this proportion. There was little effect of recruitment homophily on age and prevalent HIV as homophily was around 1 across sites.

Appendix 1a: RDS diagnostics for 2013 data

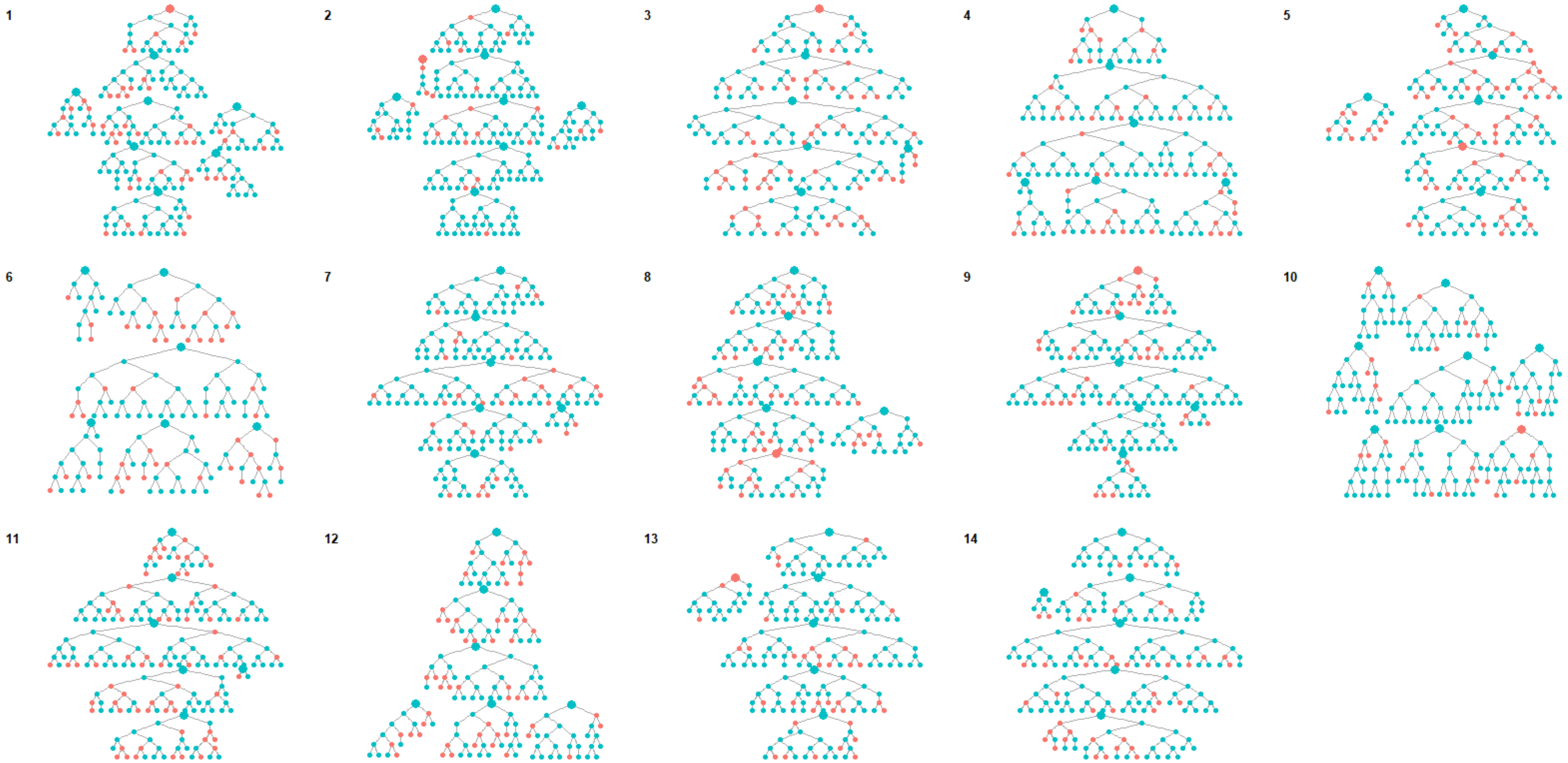

Figure 1: Rectruitment tree diagrams. Participants are depicted by circles with their recruits shown as the connected circles below them. The larger circles denote seed participants. Red circles represent women aged 18-24 and blue circles represent women aged ≥25.

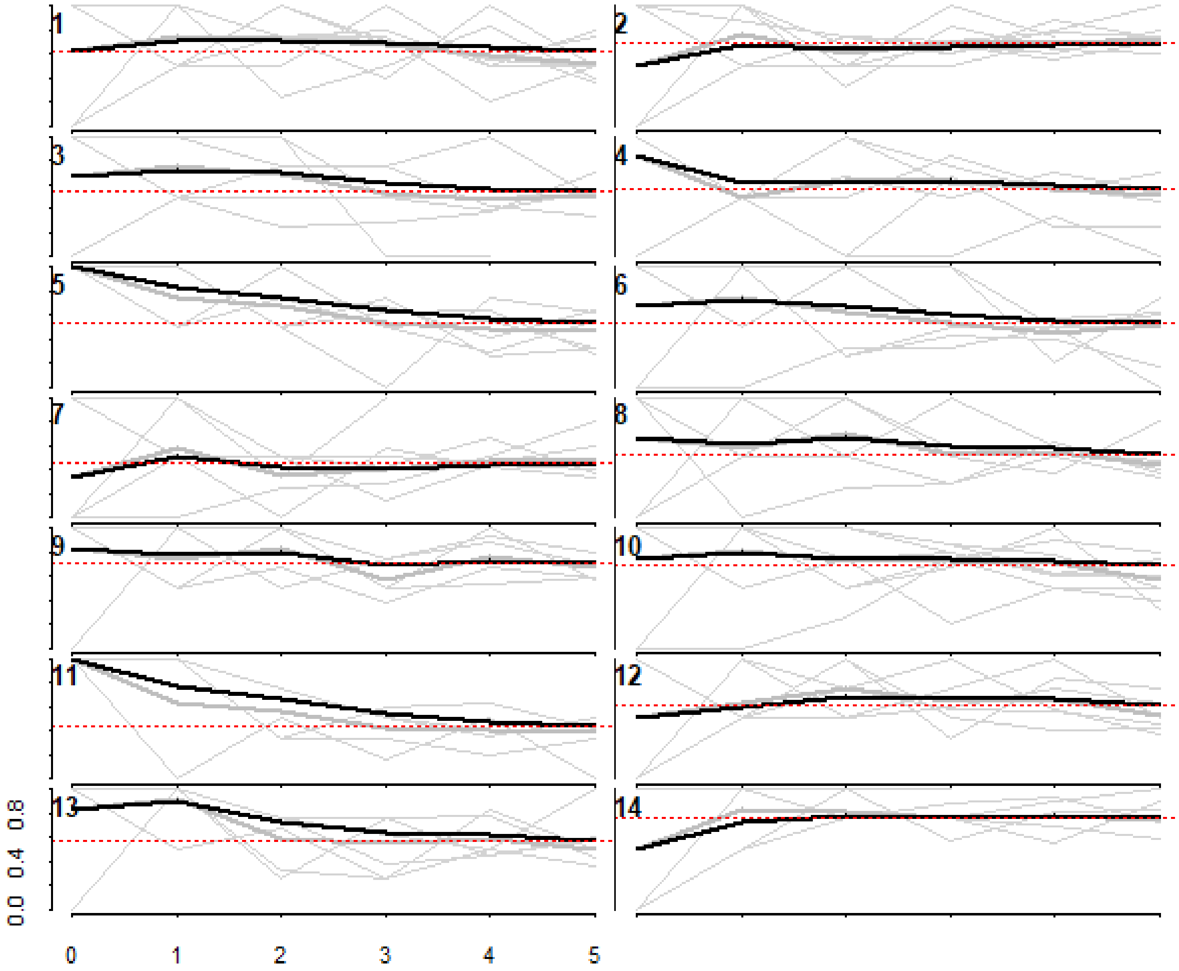

Figure 2: Convergence of the proportion of HIV positive FSW. The heavy black lines indicate the cumulative RDS-II weighted estimate overall for each site, while the grey lines are unweighted proportions for each seed, by sample wave.

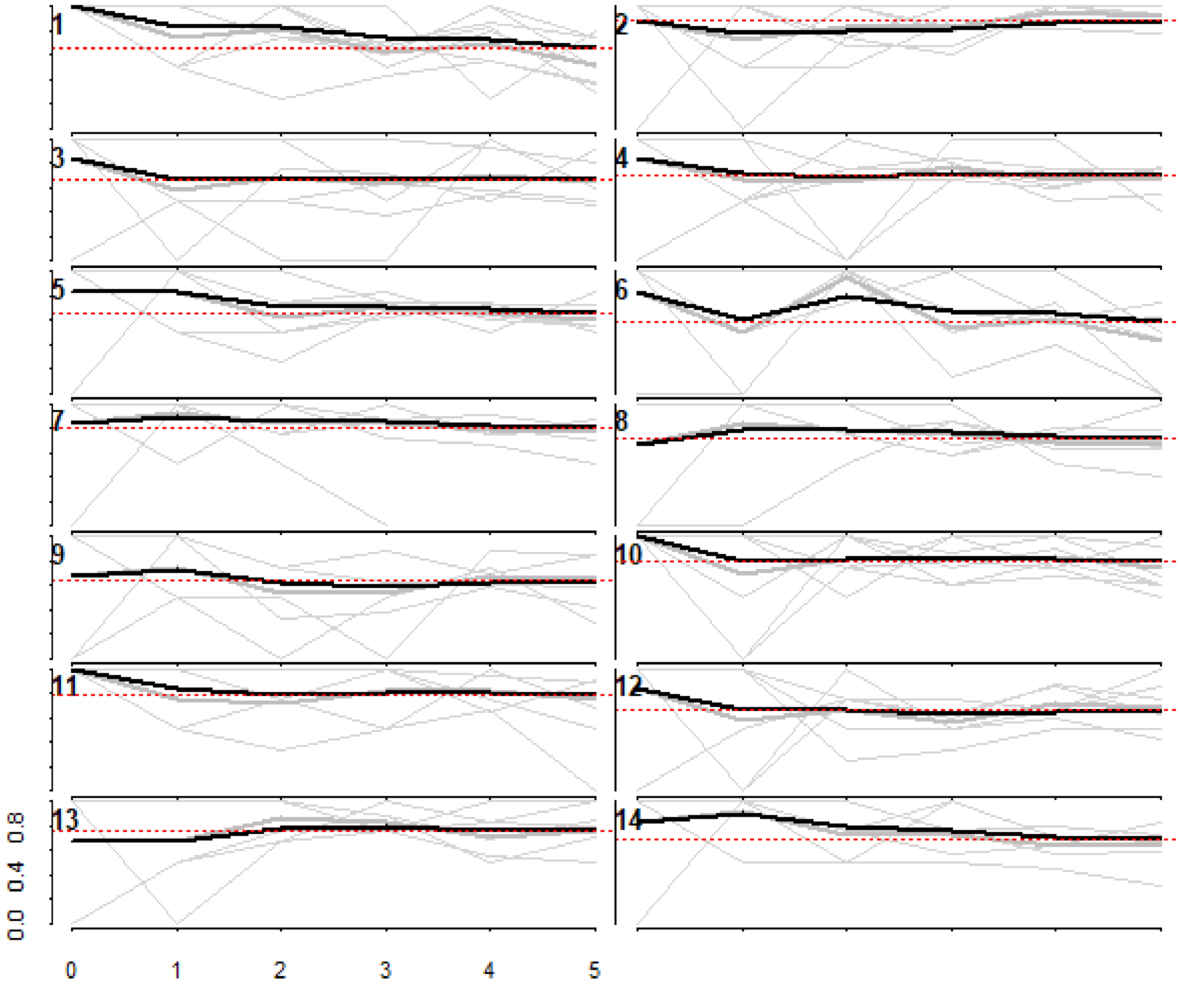

Figure 3: Convergence of the proportion of FSW who reported 3 or more years selling sex. The heavy black lines indicate the cumulative RDS-II weighted estimate overall for each site, while the grey lines are unweighted proportions for each seed, by sample wave.

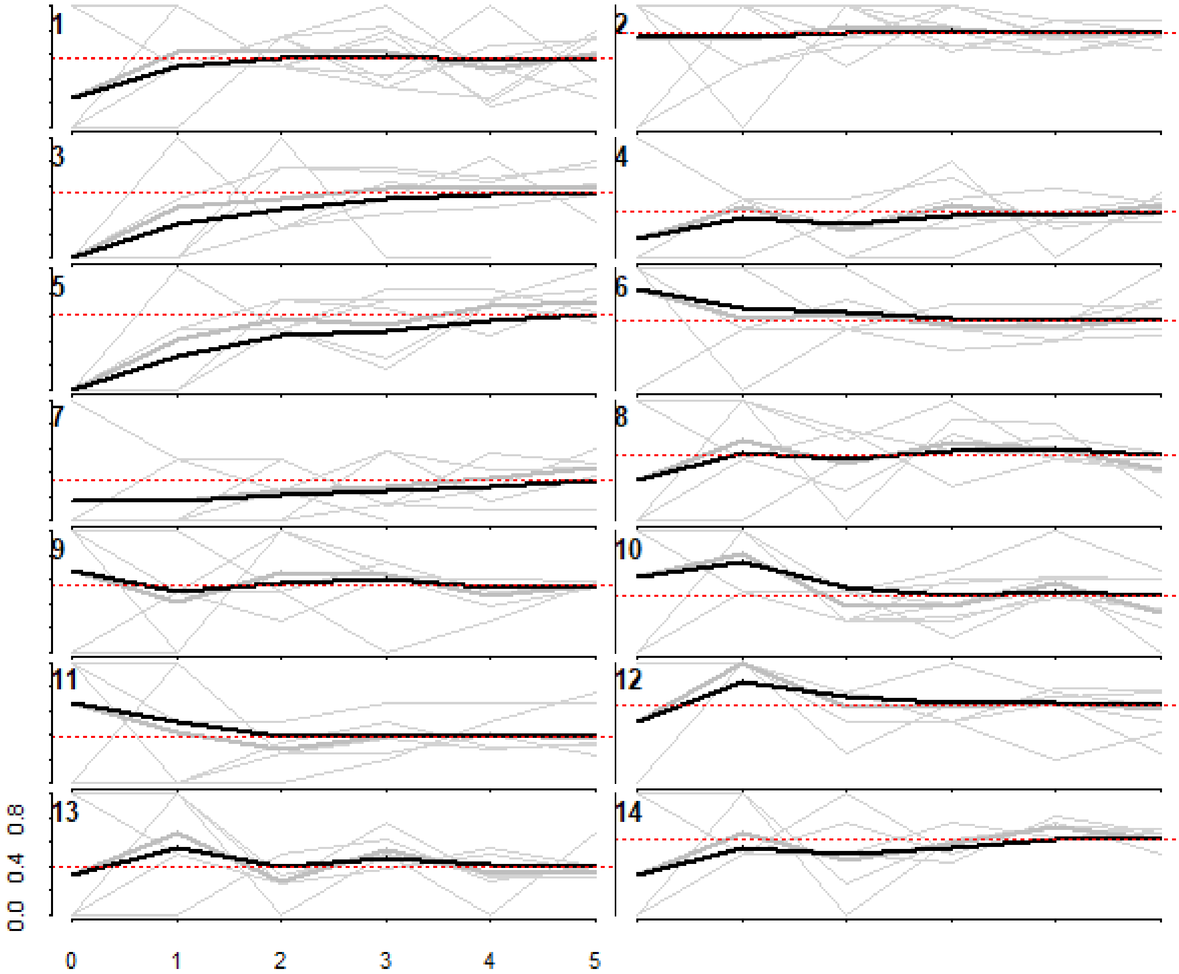

Figure 4: Convergence of the proportion of FSW who reported 5 or clients in the last week. The heavy black lines indicate the cumulative RDS-II weighted estimate overall for each site, while the grey lines are unweighted proportions for each seed, by sample wave.

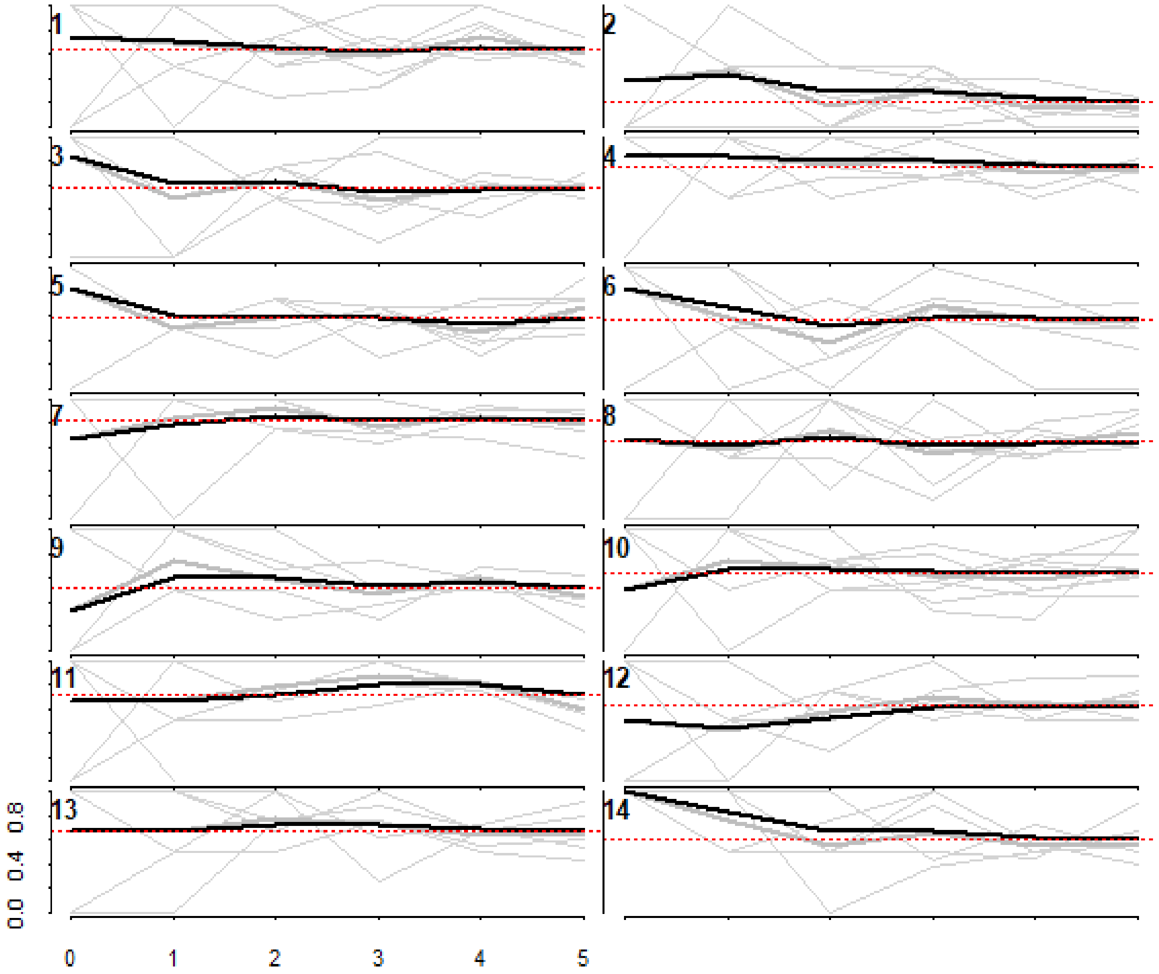

Figure 5: Convergence of the proportion of FSW who reported to have a steady partner. The heavy black lines indicate the cumulative RDS-II weighted estimate overall for each site, while the grey lines are unweighted proportions for each seed, by sample wave.

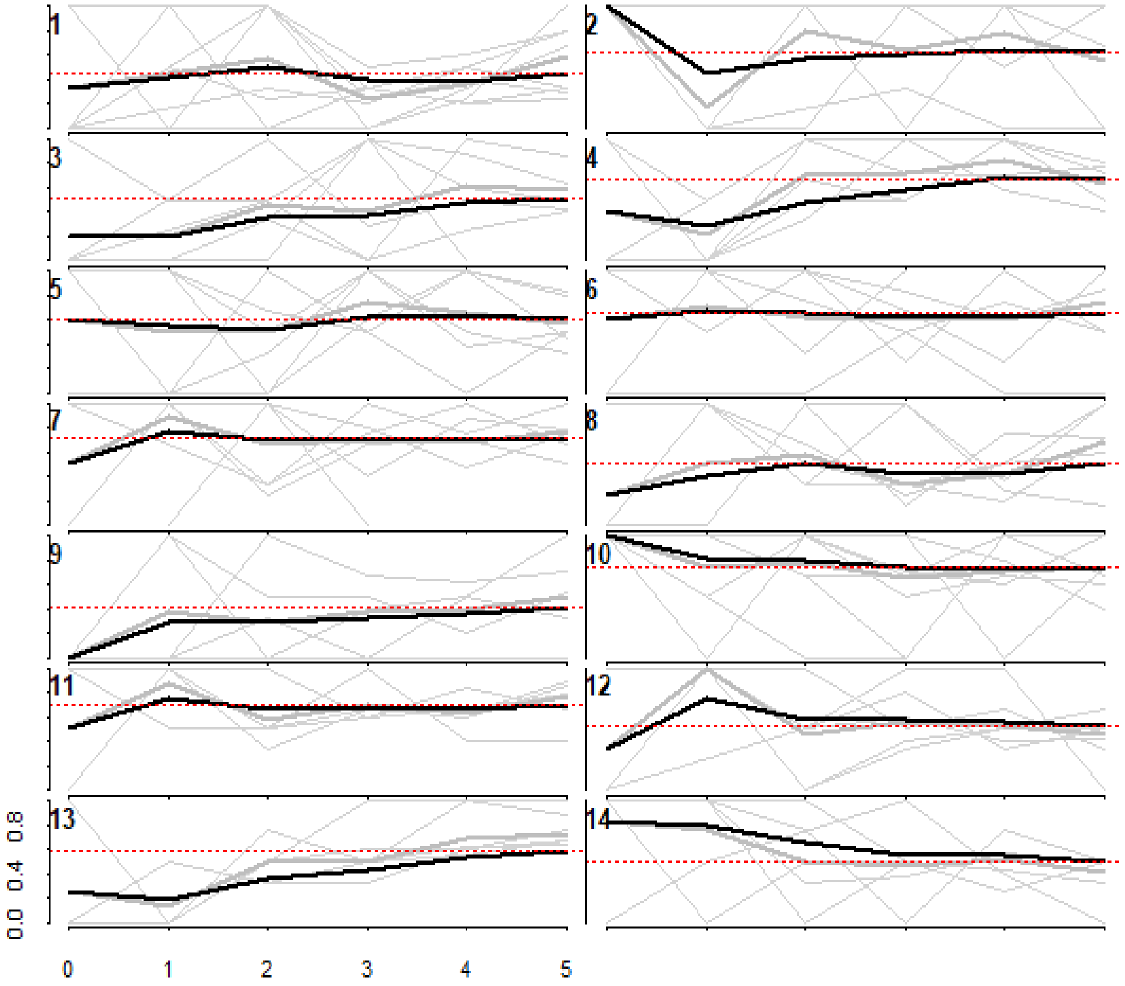

Figure 6: Convergence of the proportion of FSW who reported condom use at last sex with steady partner. The heavy black lines indicate the cumulative RDS-II weighted estimate overall for each site, while the grey lines are unweighted proportions for each seed, by sample wave.

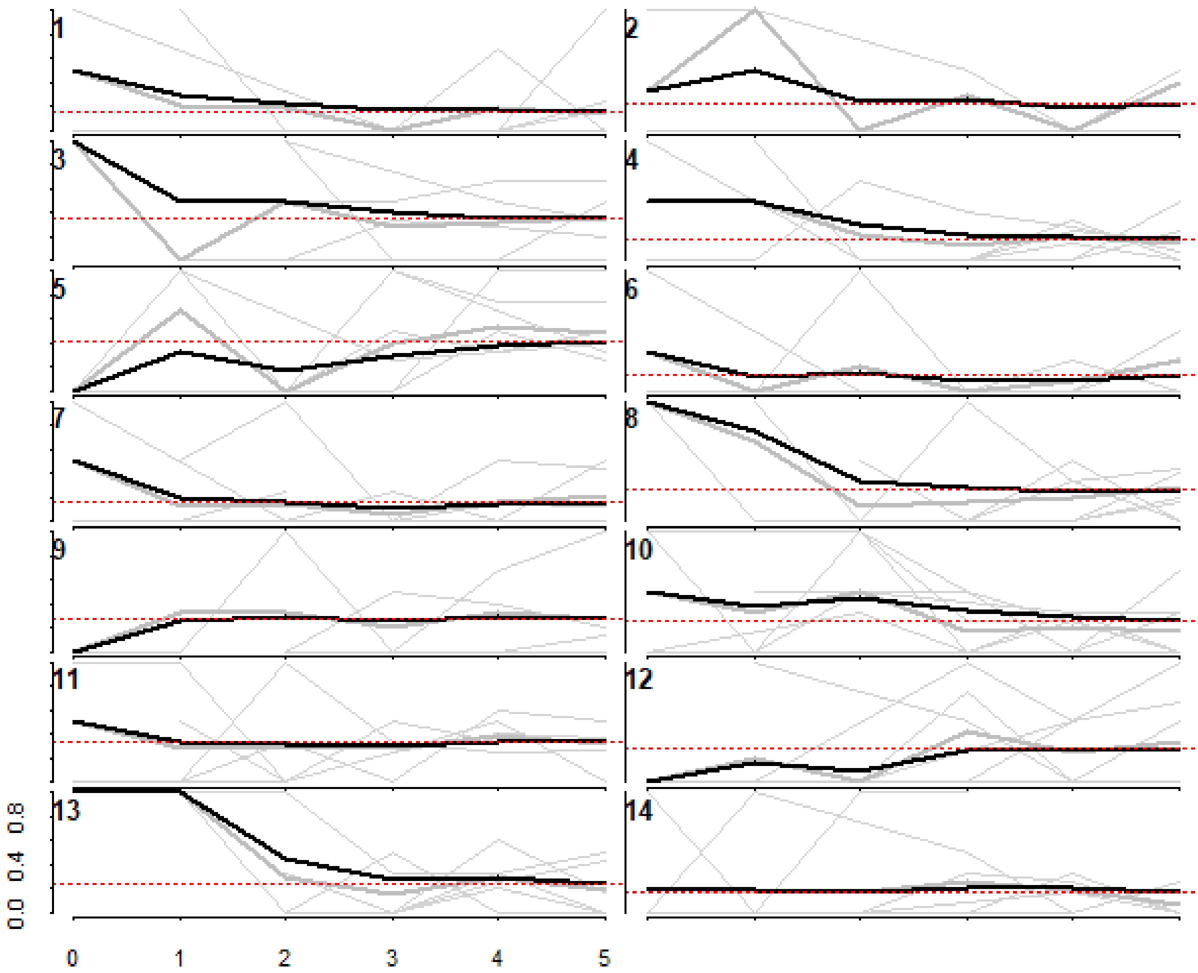

Figure 7: Convergence of the proportion of FSW who reported condom-less sex with steady partner. The heavy black lines indicate the cumulative RDS-II weighted estimate overall for each site, while the grey lines are unweighted proportions for each seed, by sample wave.

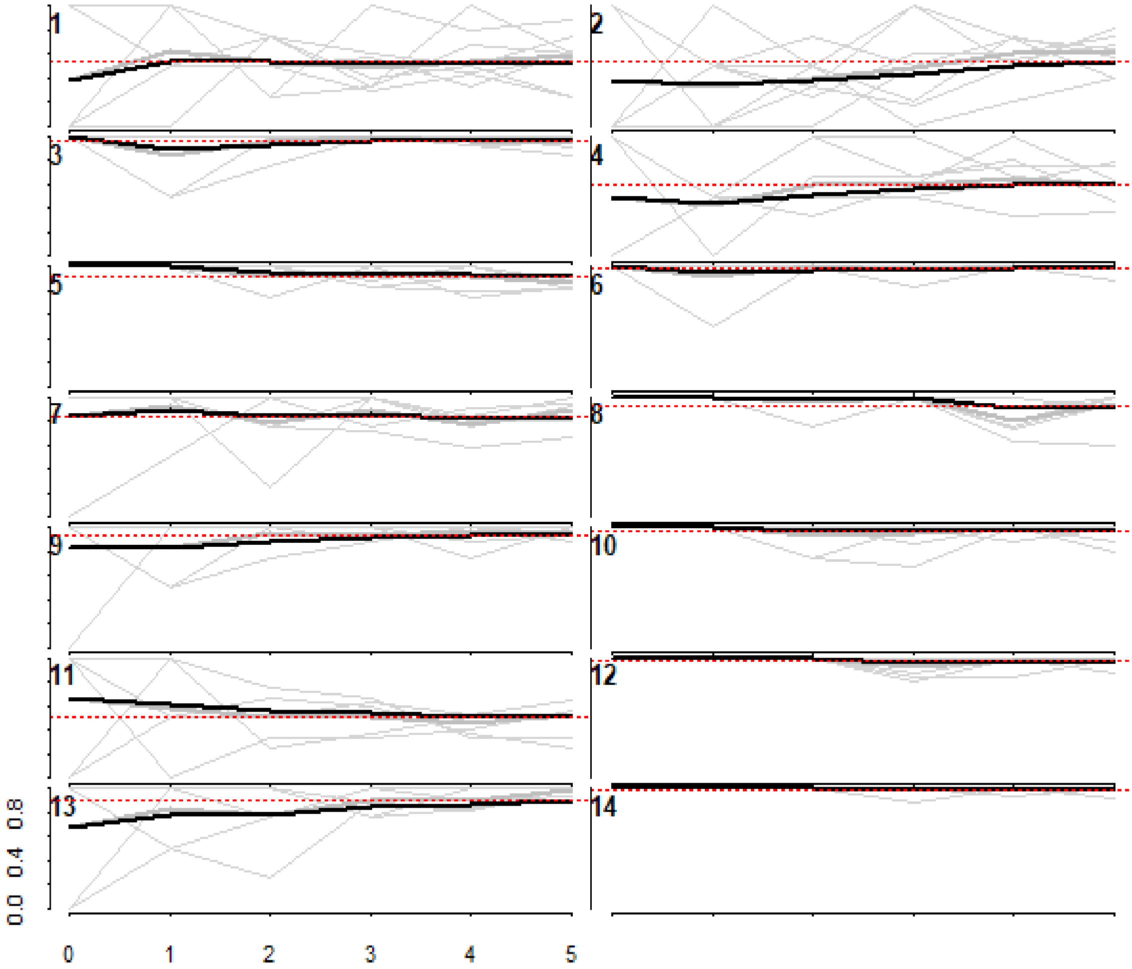

Figure 8: Convergence of the proportion of FSW who reported condom use at last sex with client. The heavy black lines indicate the cumulative RDS-II weighted estimate overall for each site, while the grey lines are unweighted proportions for each seed, by sample wave.

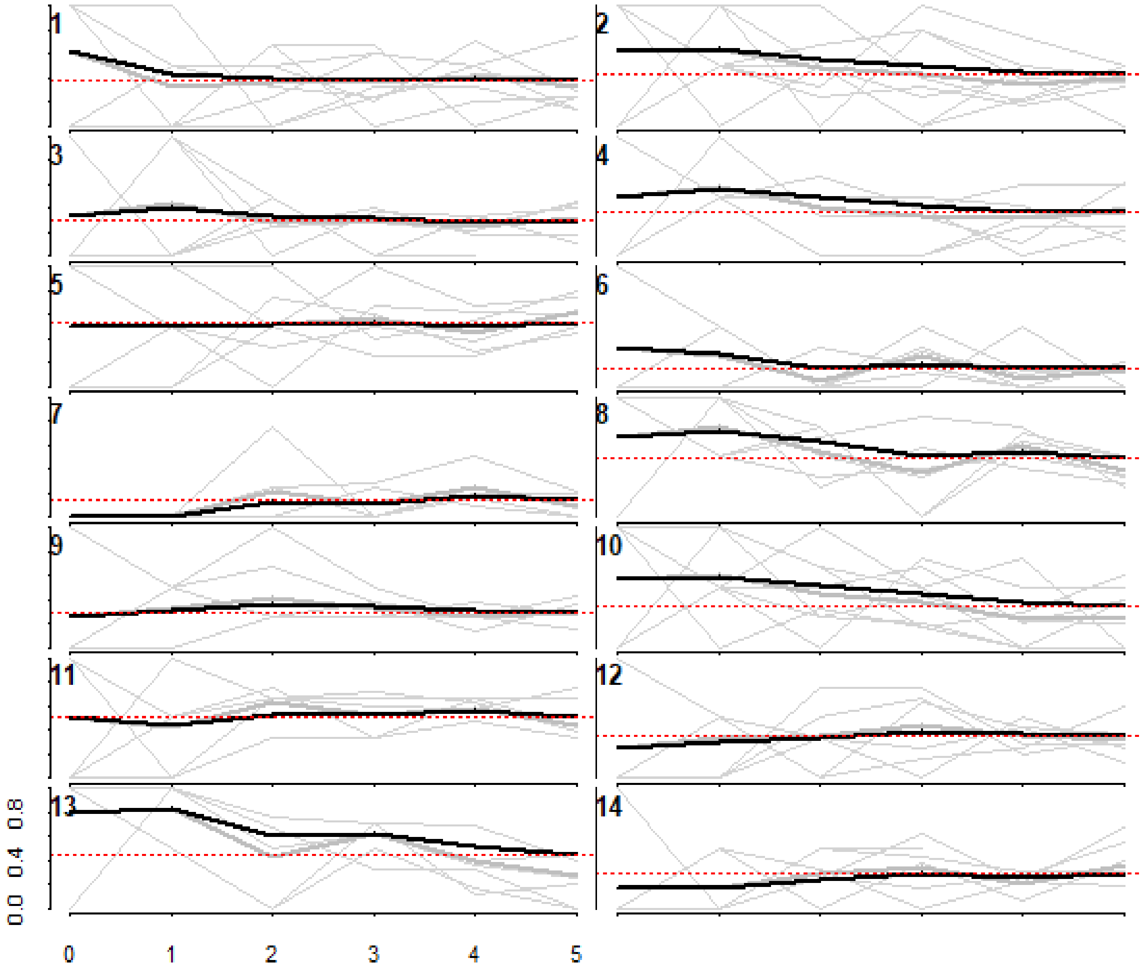

Figure 9: Convergence of the proportion of FSW who reported condom-less sex with client. The heavy black lines indicate the cumulative RDS-II weighted estimate overall for each site, while the grey lines are unweighted proportions for each seed, by sample wave.

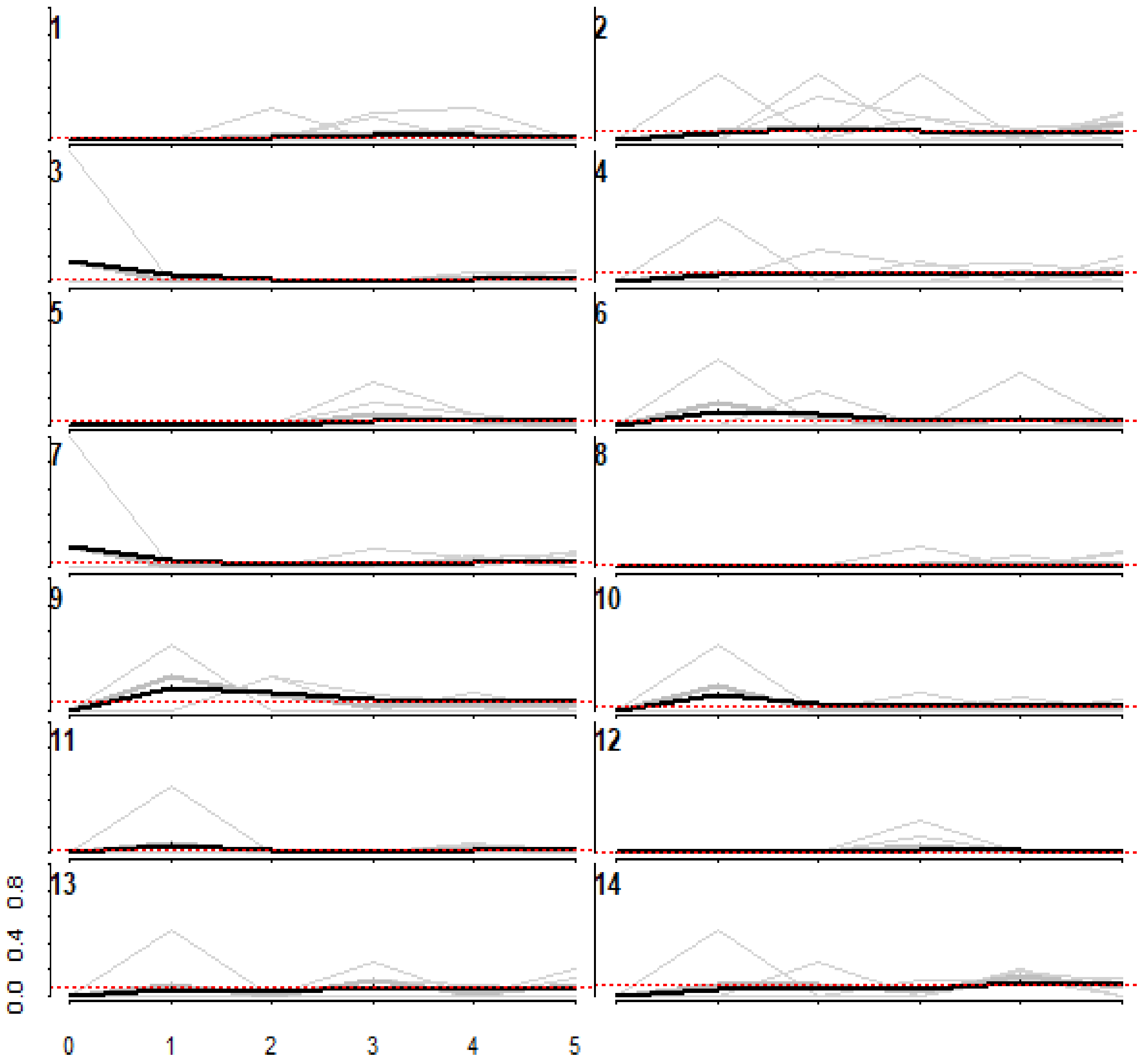

Figure 10: Convergence of the proportion of FSW who reported to ever been forced to have sexual intercourse. The heavy black lines indicate the cumulative RDS-II weighted estimate overall for each site, while the grey lines are unweighted proportions for each seed, by sample wave.

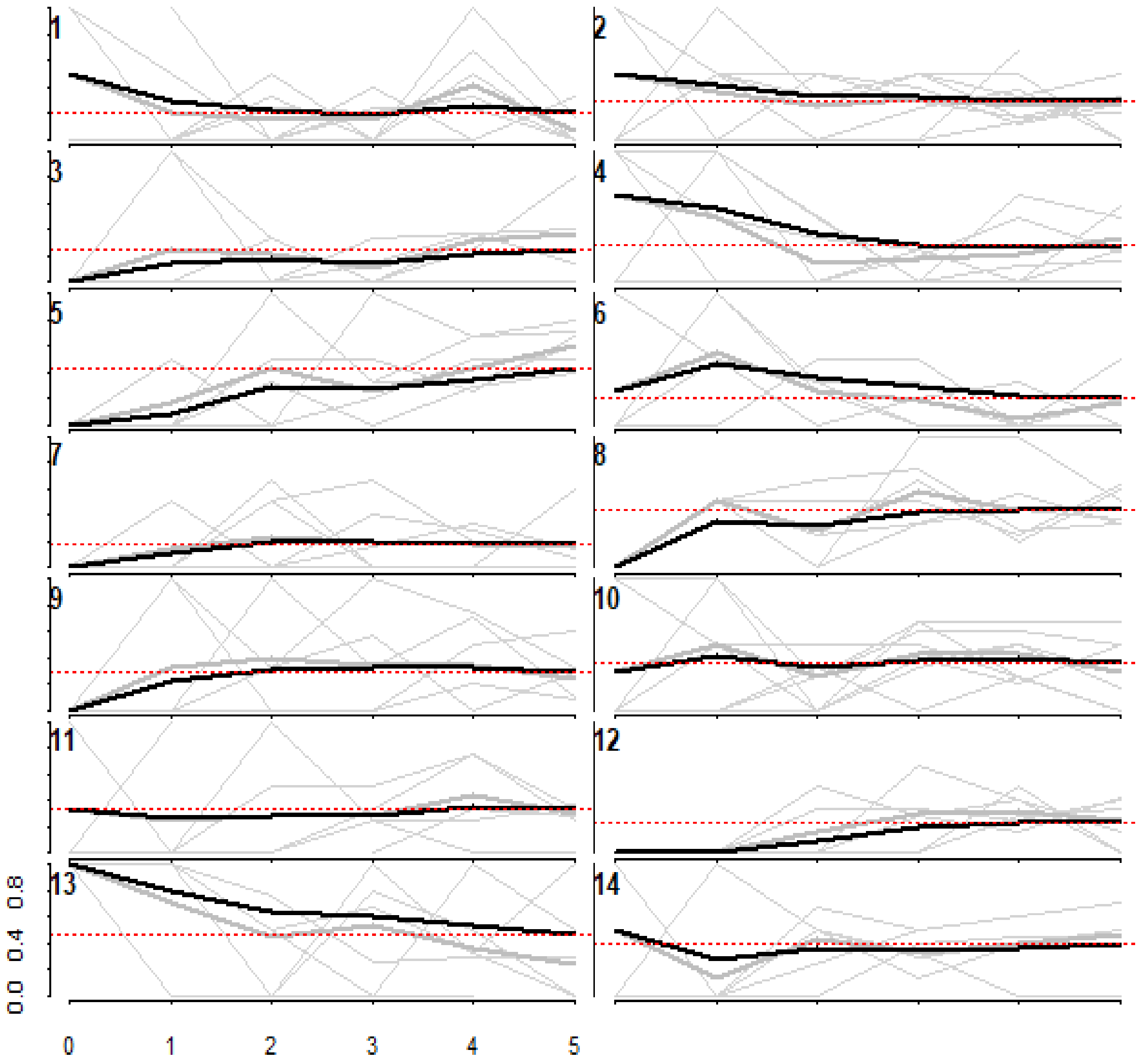

Figure 11: Convergence of the proportion of FSW who reported to have failed to use a condom with client as a result of own drinking during the past 12 months. The heavy black lines indicate the cumulative RDS-II weighted estimate overall for each site, while the grey lines are unweighted proportions for each seed, by sample wave.

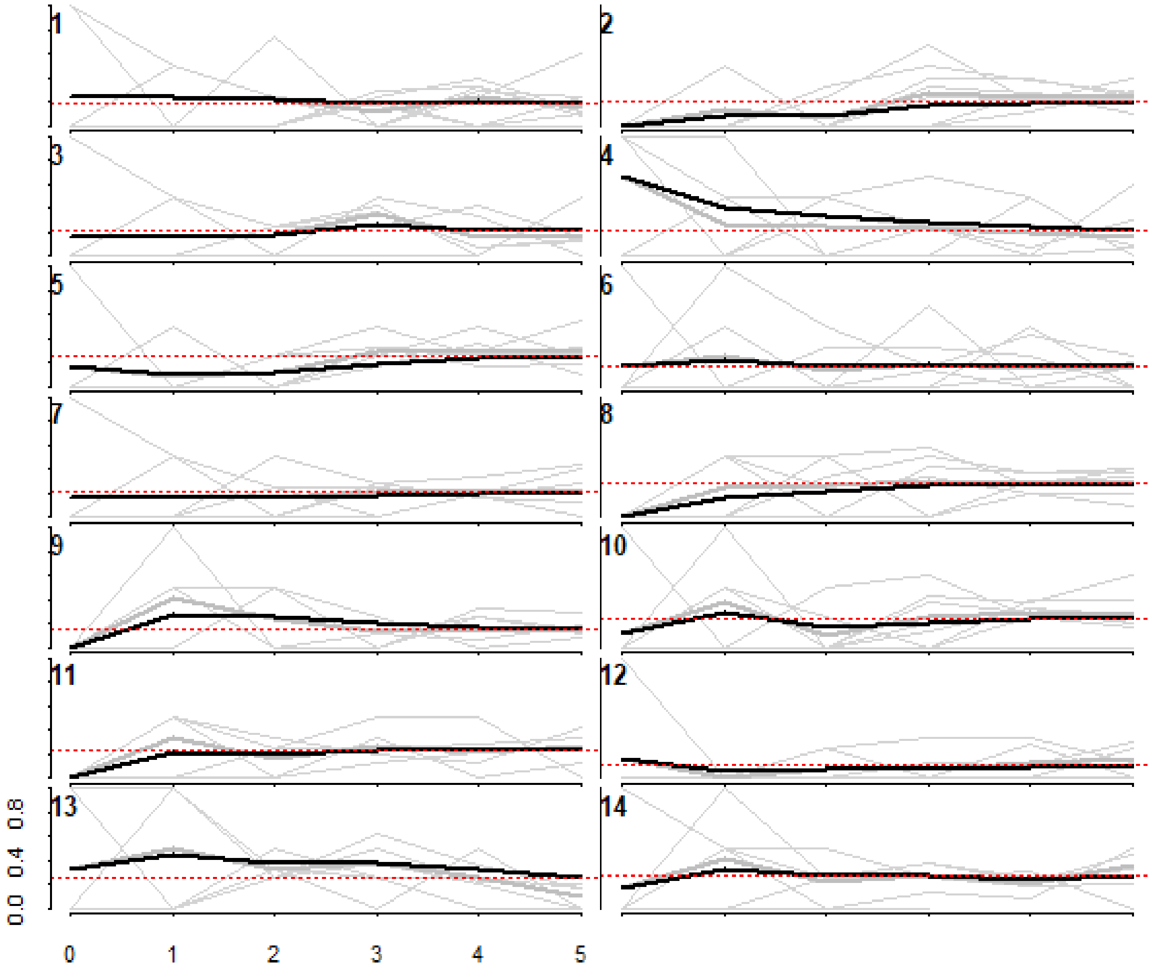

Figure 12: Convergence of the proportion of FSW who reported to have failed to use a condom with a client as a result of client's drinking during the past 12 months. The heavy black lines indicate the cumulative RDS-II weighted estimate overall for each site, while the grey lines are unweighted proportions for each seed, by sample wave.

Appendix 1b: RDS diagnostics for 2016 data

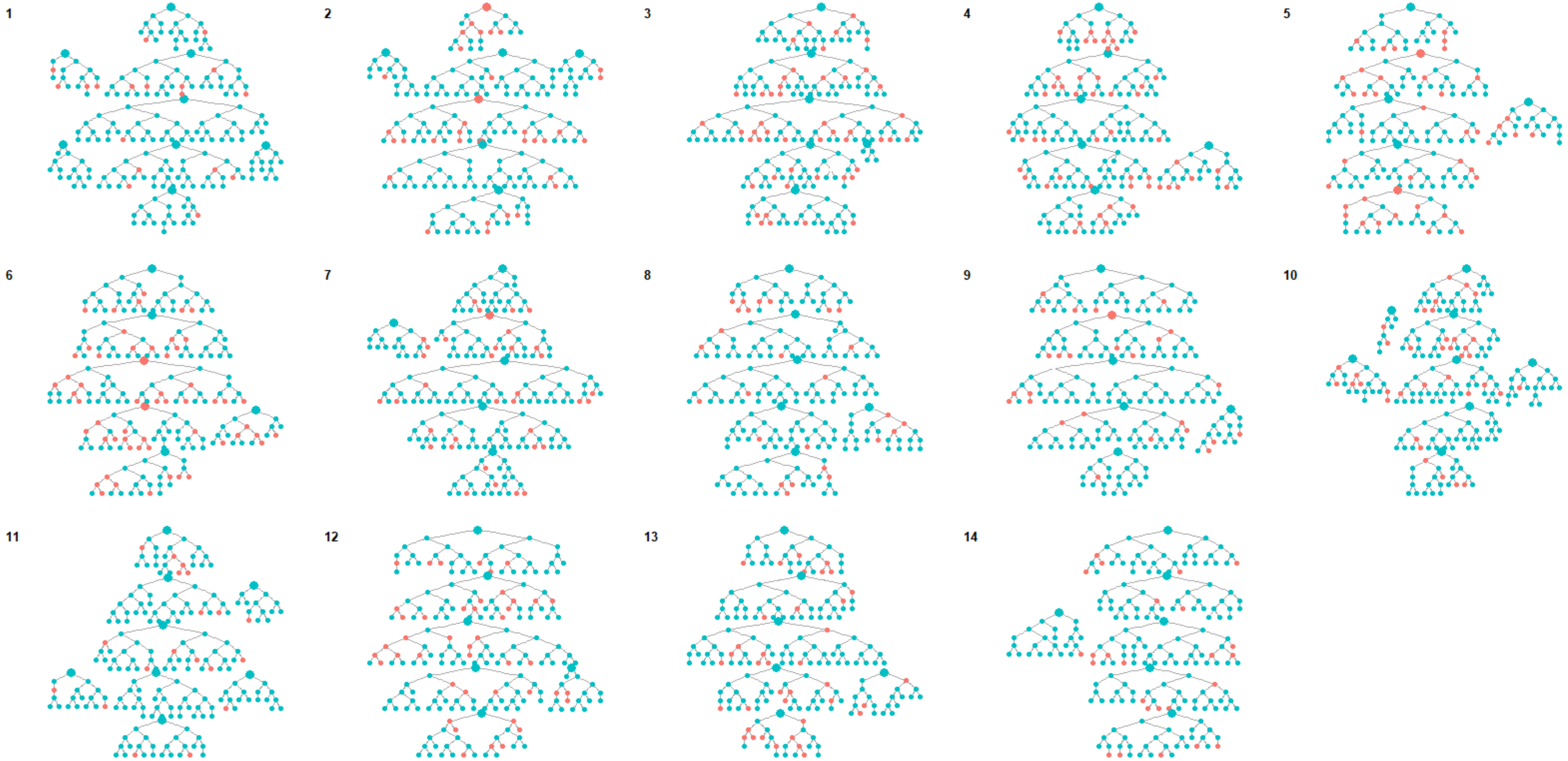

Figure 13: Rectruitment tree diagrams. Participants are depicted by circles with their recruits shown as the connected circles below them. The larger circles denote seed participants. Red circles represent women aged 18-24 and blue circles represent women aged ≥25.

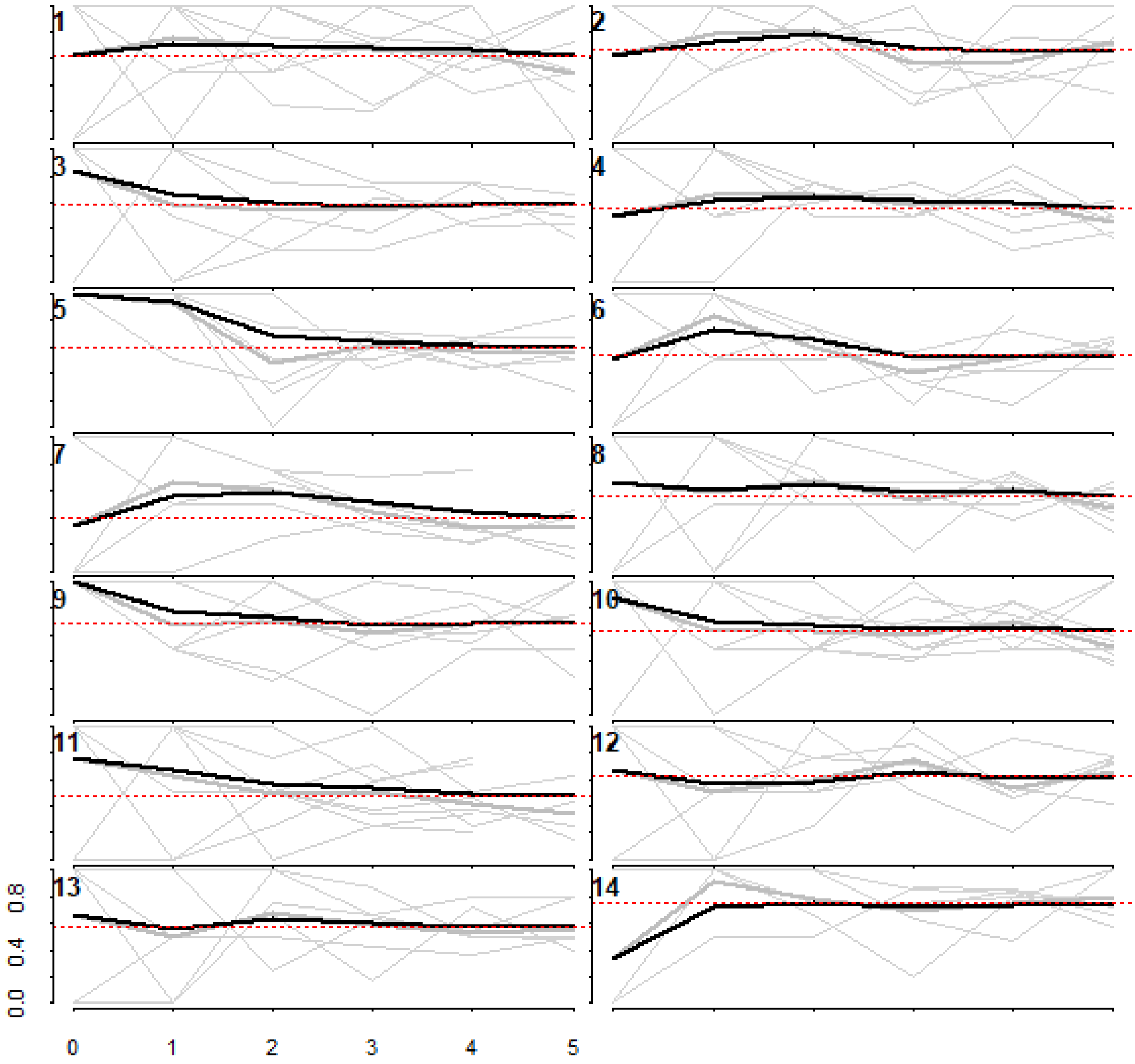

Figure 14: Convergence of the proportion of HIV positive FSW. The heavy black lines indicate the cumulative RDS-II weighted estimate overall for each site, while the grey lines are unweighted proportions for each seed, by sample wave.

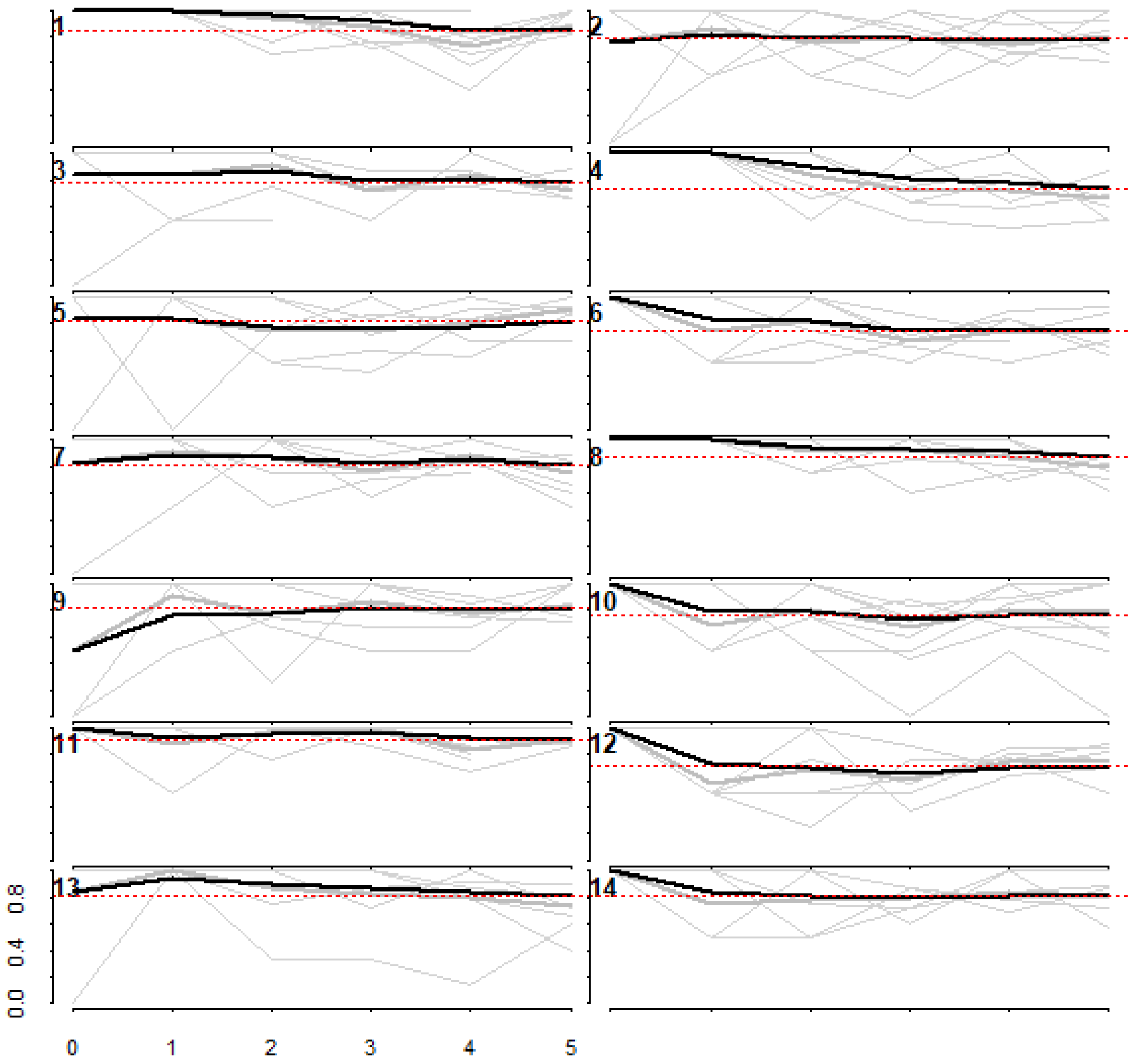

Figure 15: Convergence of the proportion of FSW who reported 3 or more years selling sex. The heavy black lines indicate the cumulative RDS-II weighted estimate overall for each site, while the grey lines are unweighted proportions for each seed, by sample wave.

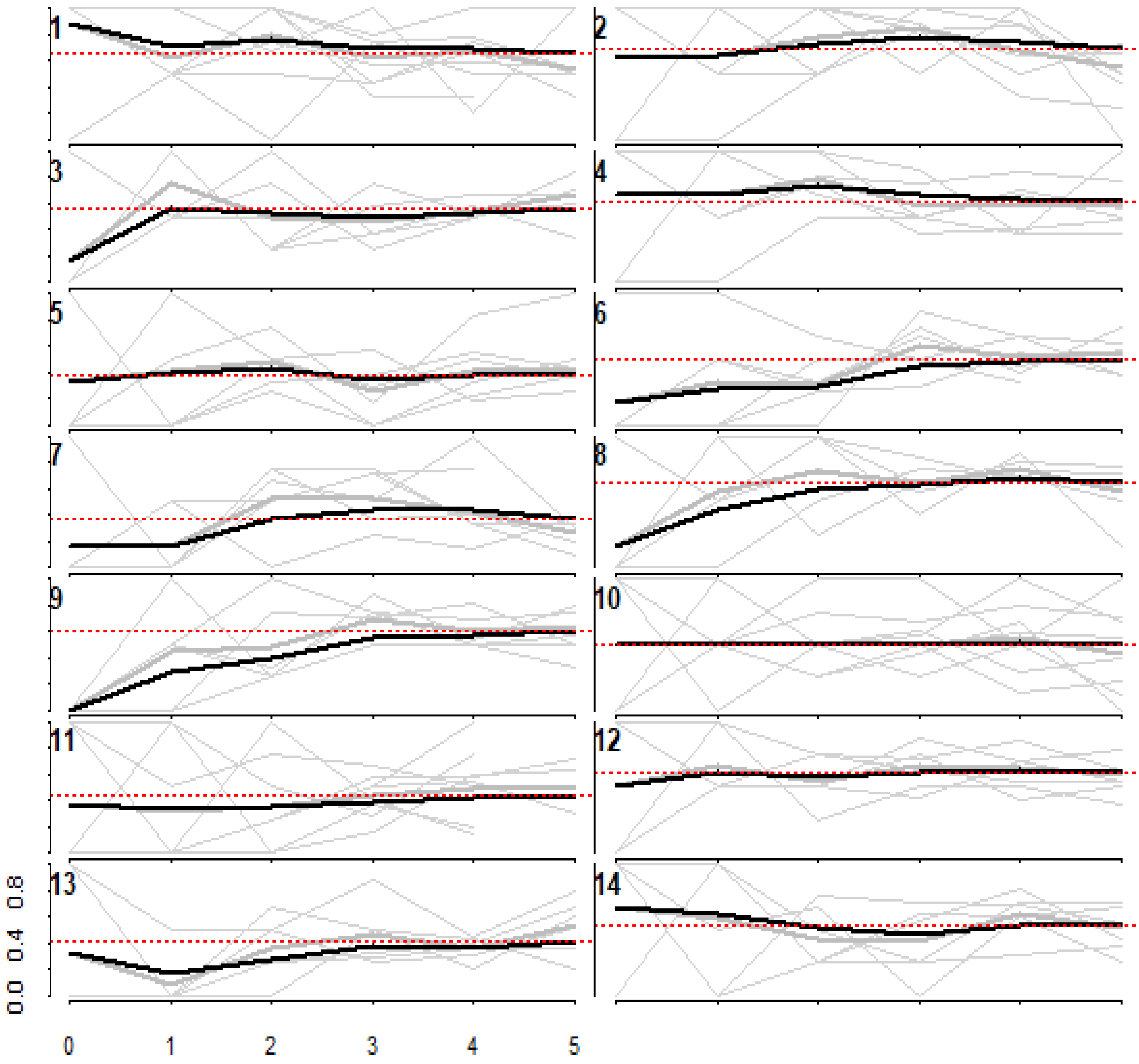

Figure 16: Convergence of the proportion of FSW who reported 5 or clients in the last week. The heavy black lines indicate the cumulative RDS-II weighted estimate overall for each site, while the grey lines are unweighted proportions for each seed, by sample wave.

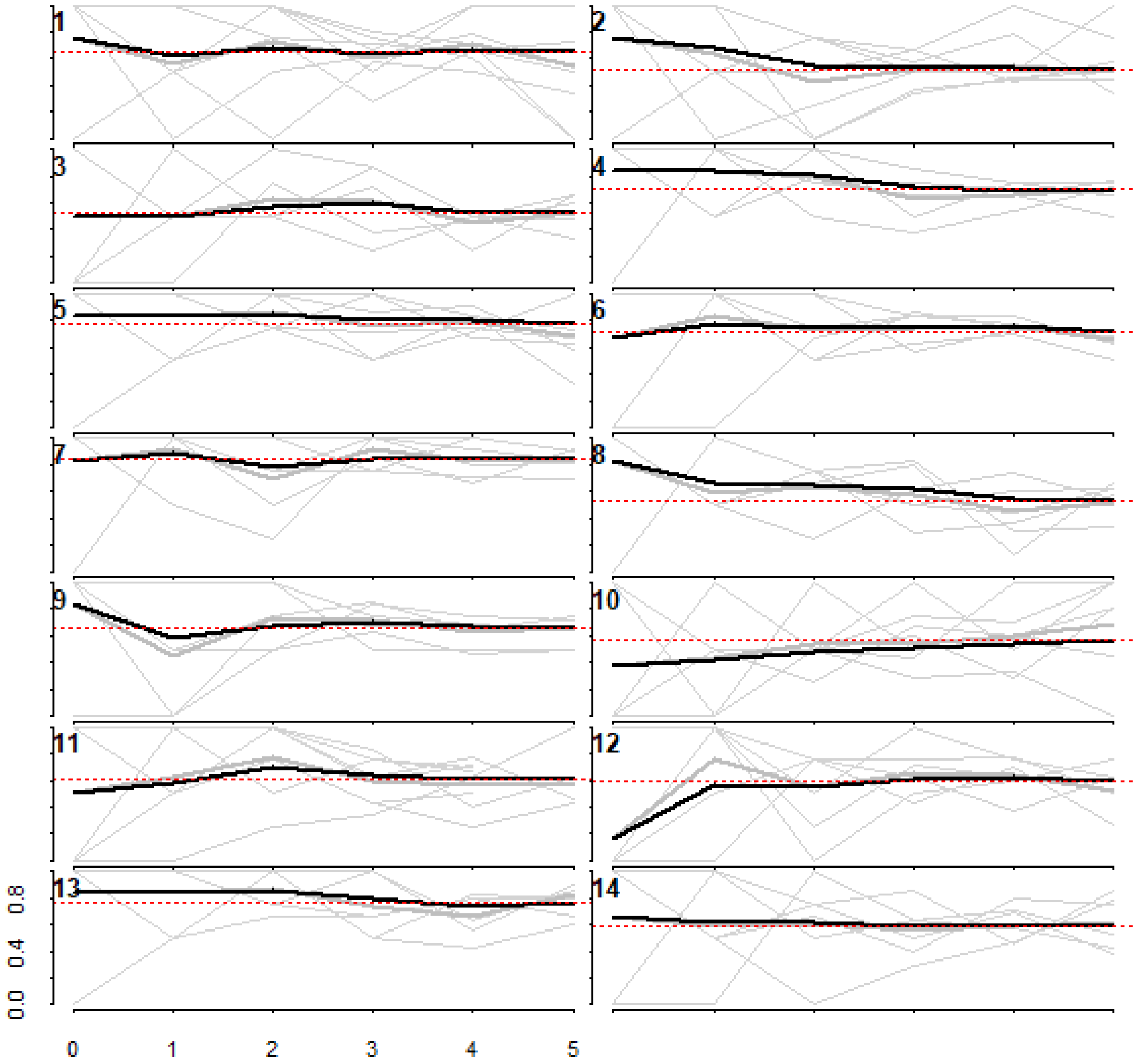

Figure 17: Convergence of the proportion of FSW who reported to have a steady partner. The heavy black lines indicate the cumulative RDS-II weighted estimate overall for each site, while the grey lines are unweighted proportions for each seed, by sample wave.

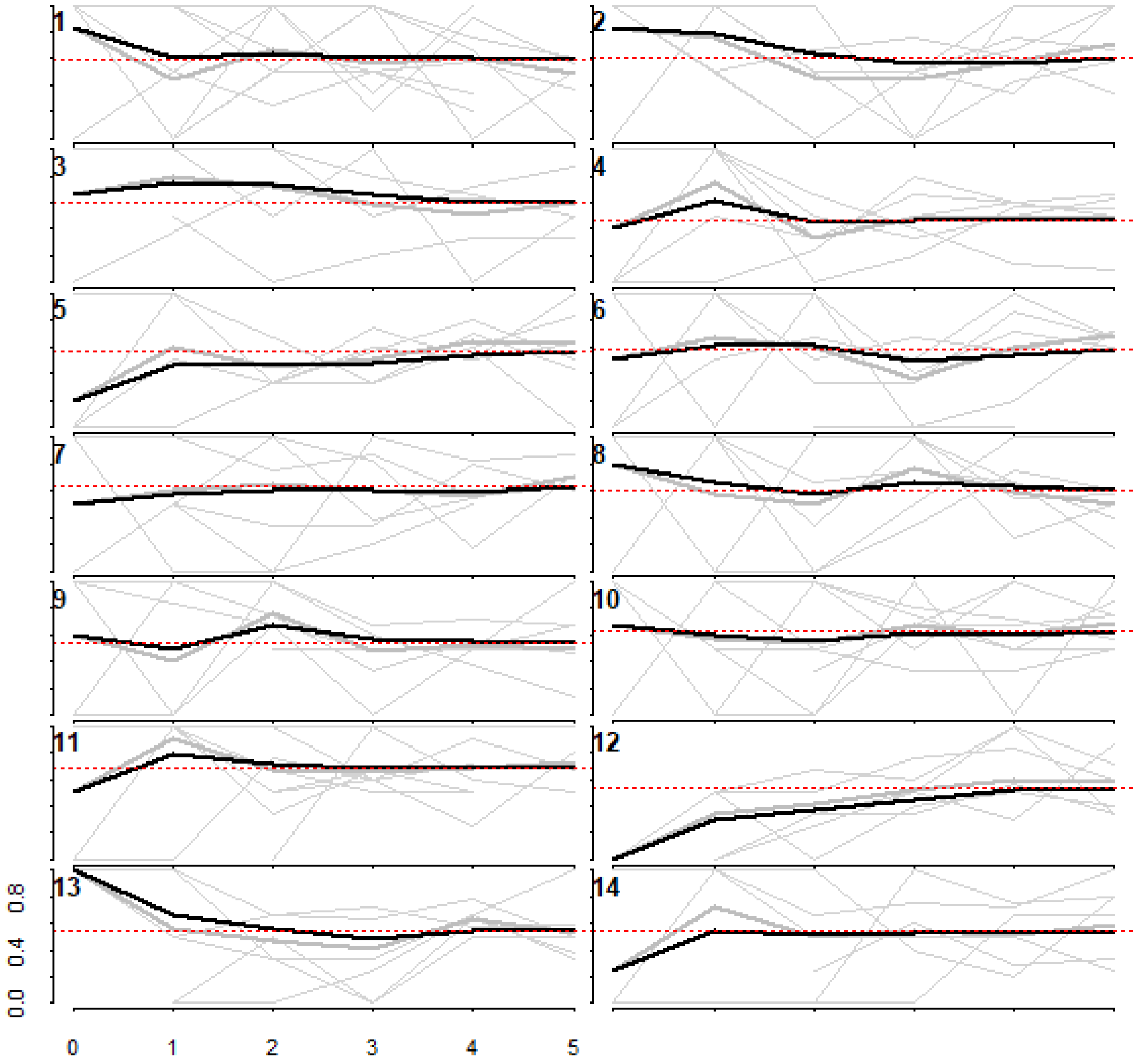

Figure 18: Convergence of the proportion of FSW who reported condom use at last sex with steady partner. The heavy black lines indicate the cumulative RDS-II weighted estimate overall for each site, while the grey lines are unweighted proportions for each seed, by sample wave.

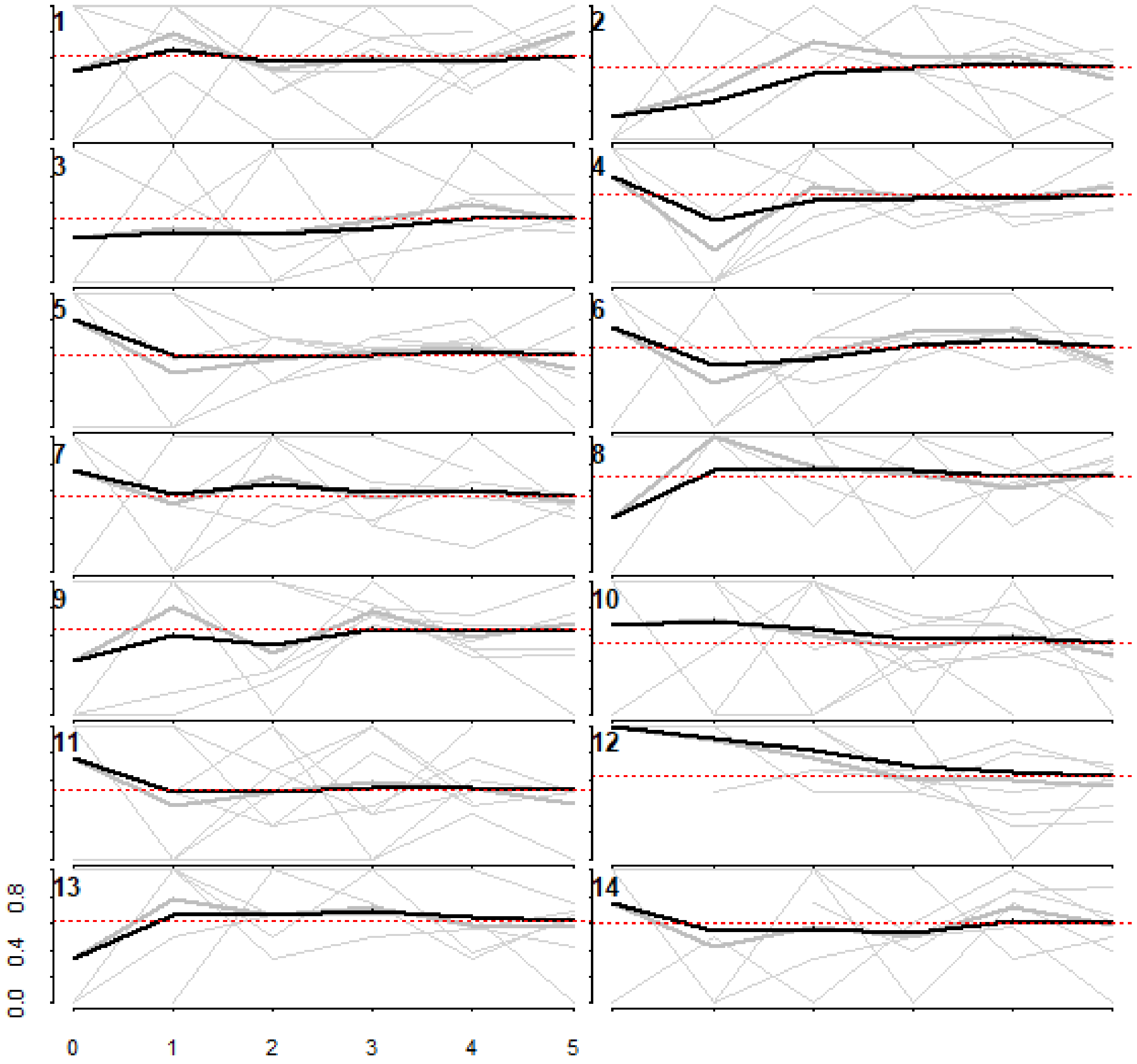

Figure 19: Convergence of the proportion of FSW who reported condom-less sex with steady partner. The heavy black lines indicate the cumulative RDS-II weighted estimate overall for each site, while the grey lines are unweighted proportions for each seed, by sample wave.

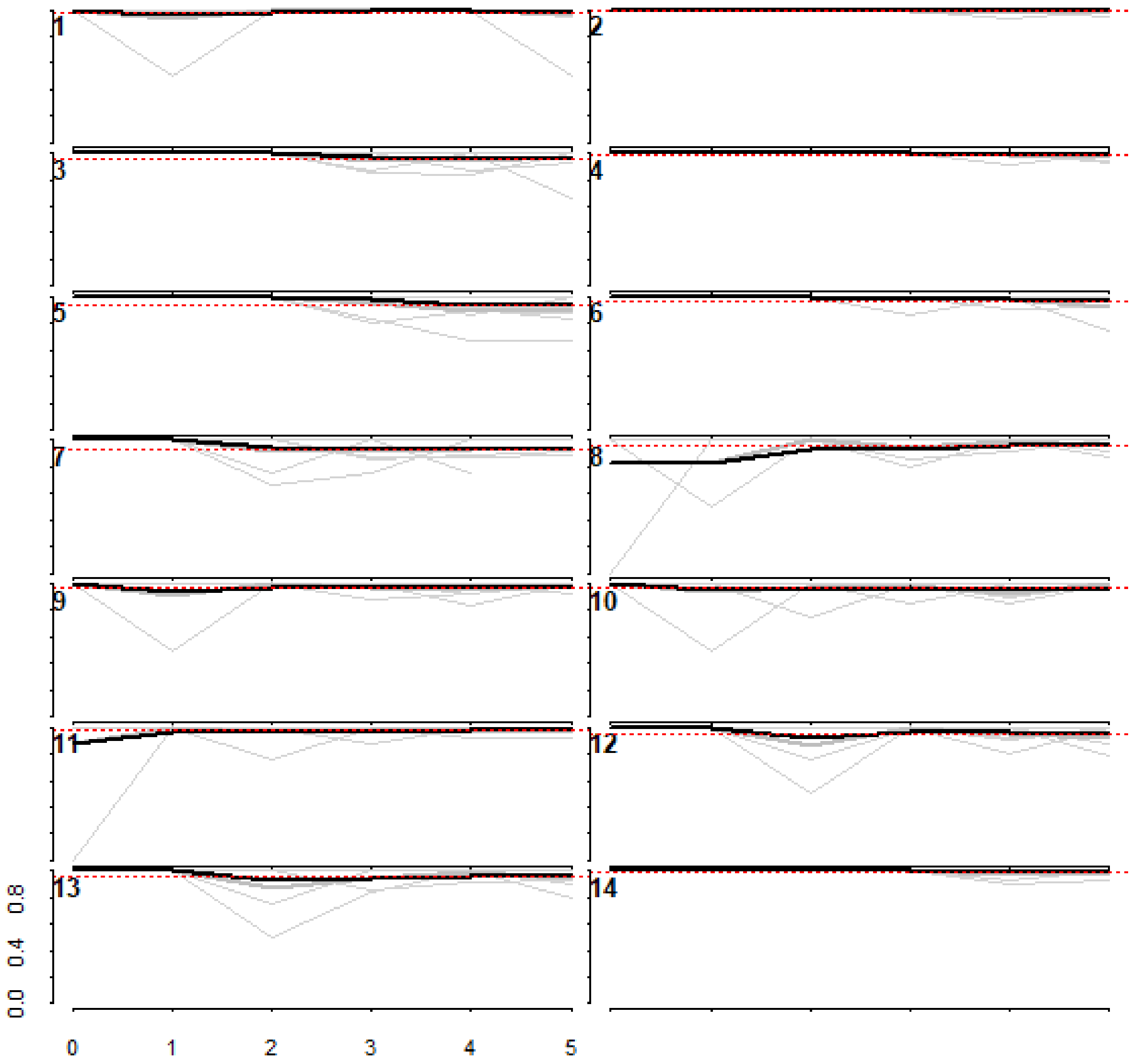

Figure 20: Convergence of the proportion of FSW who reported condom use at last sex with client. The heavy black lines indicate the cumulative RDS-II weighted estimate overall for each site, while the grey lines are unweighted proportions for each seed, by sample wave.

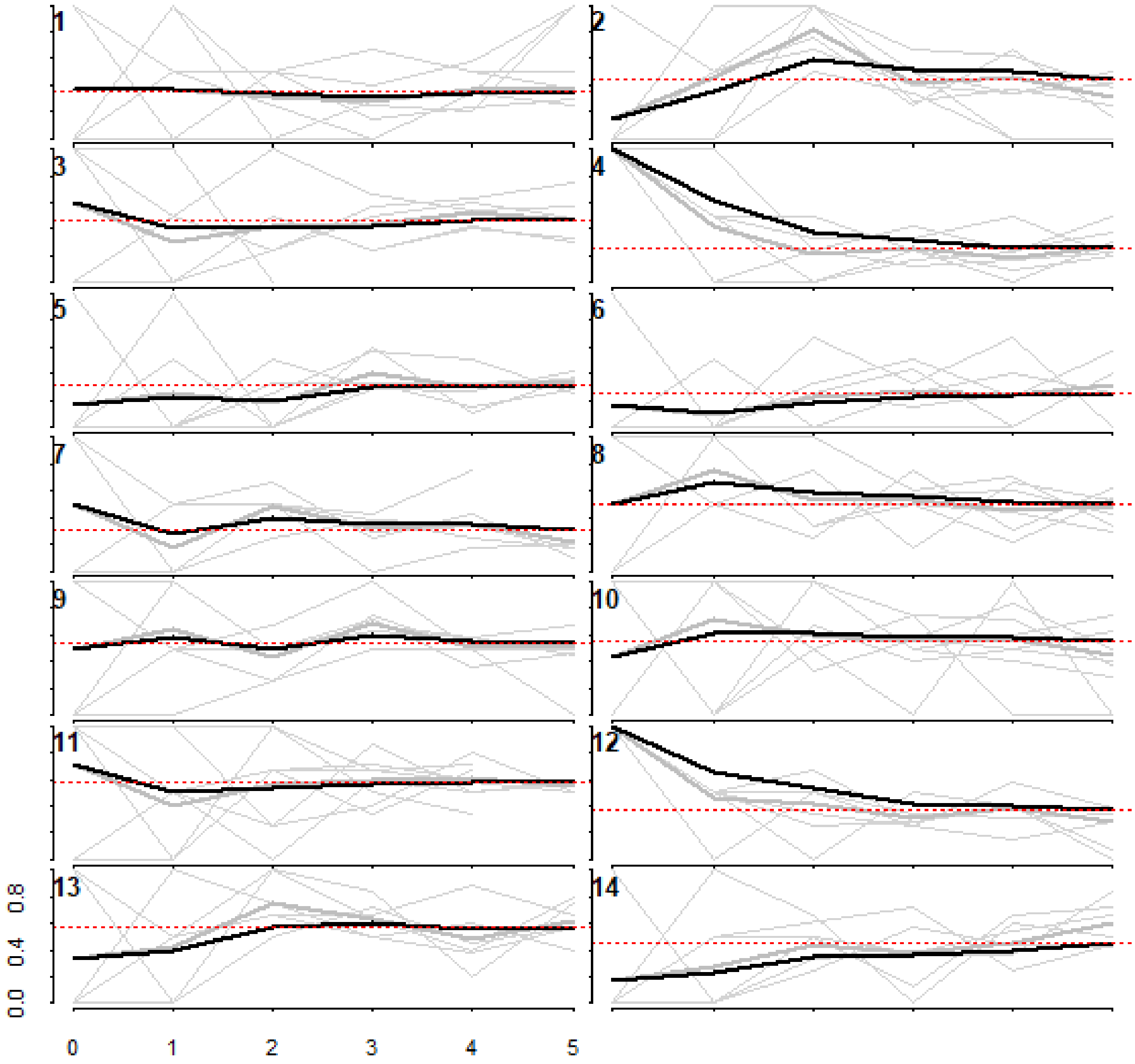

Figure 21: Convergence of the proportion of FSW who reported condom-less sex with client. The heavy black lines indicate the cumulative RDS-II weighted estimate overall for each site, while the grey lines are unweighted proportions for each seed, by sample wave.

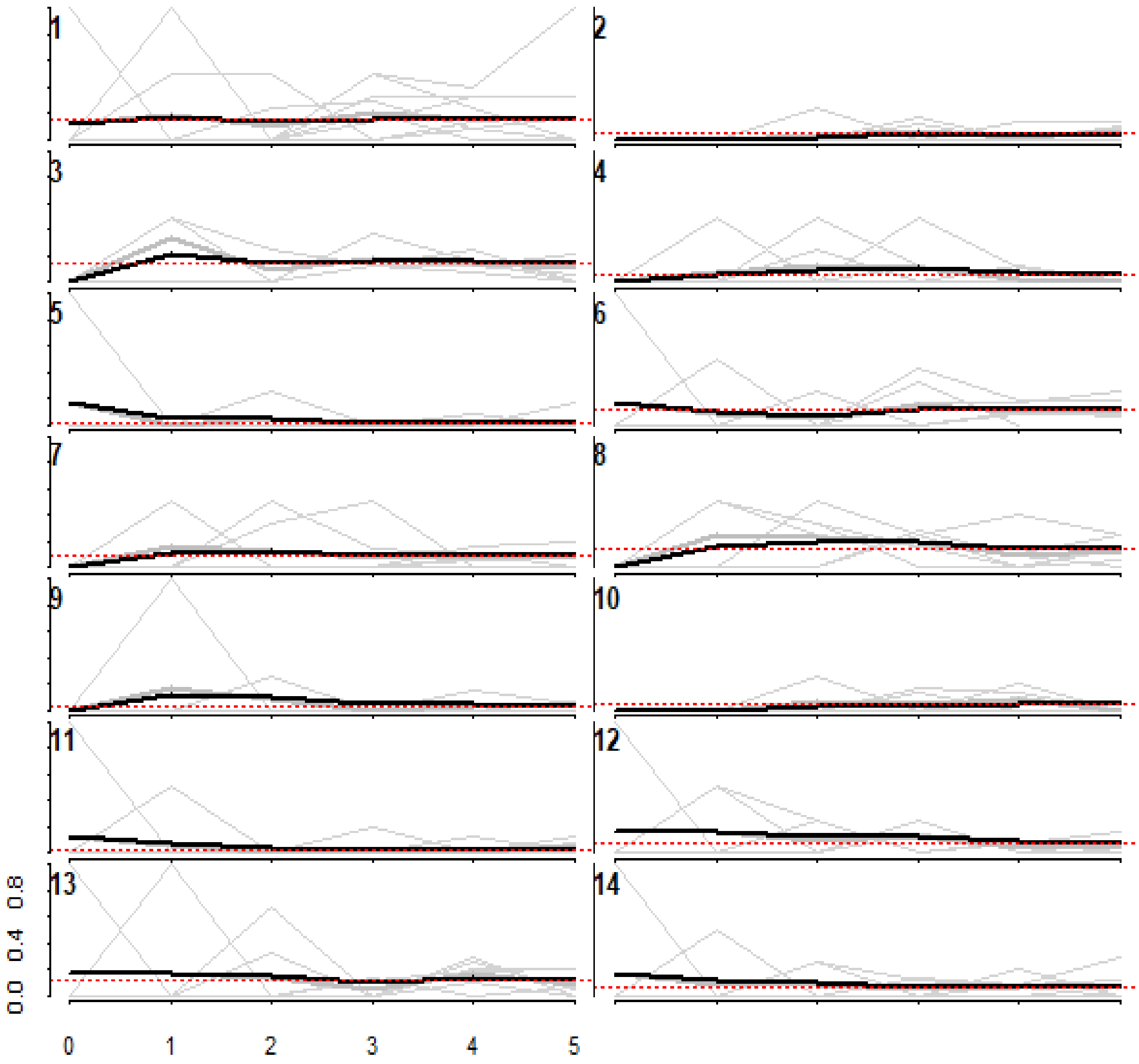

Figure 22: Convergence of the proportion of FSW who reported to ever been forced to have sexual intercourse. The heavy black lines indicate the cumulative RDS-II weighted estimate overall for each site, while the grey lines are unweighted proportions for each seed, by sample wave.

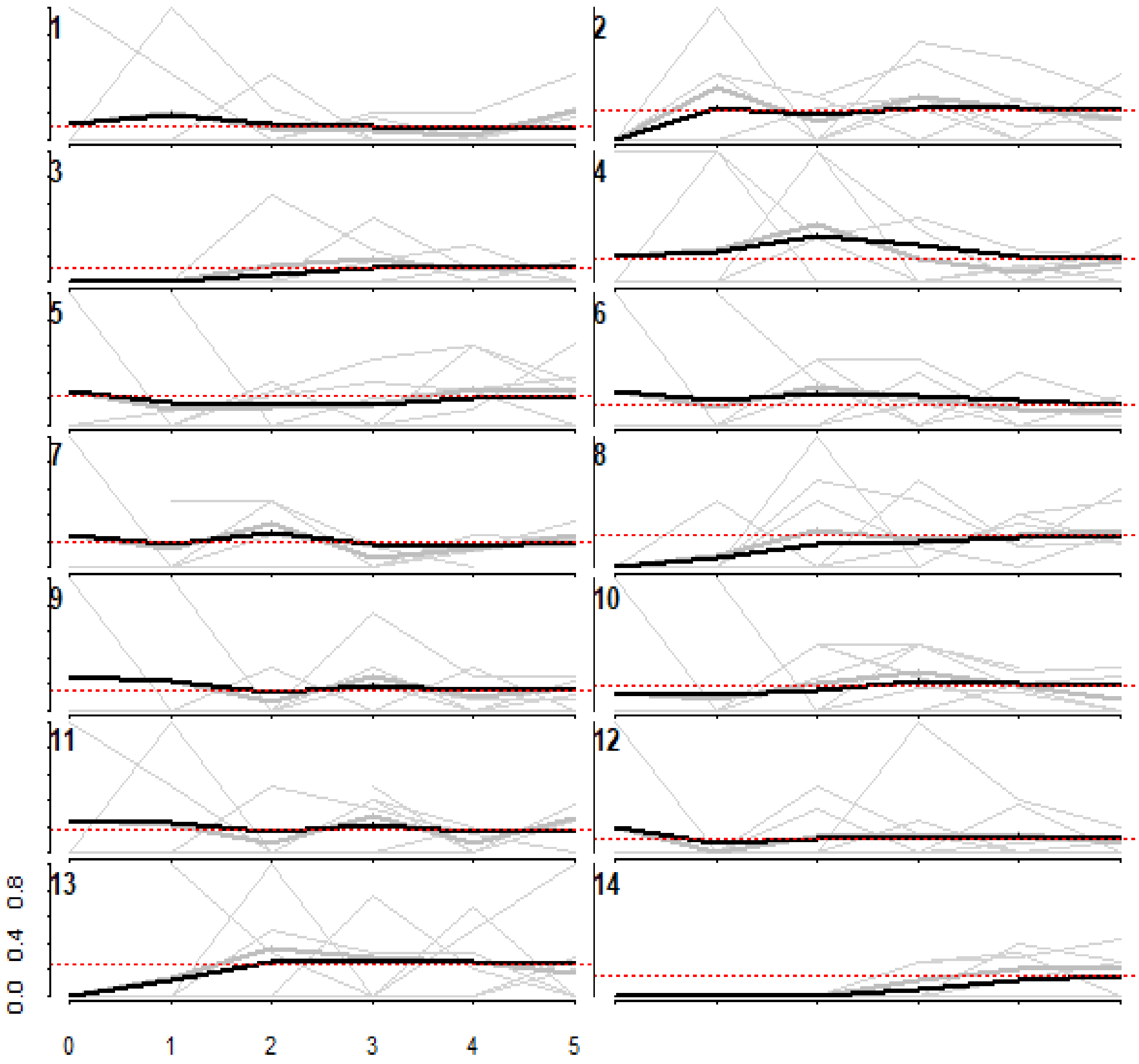

Figure 23: Convergence of the proportion of FSW who reported to have failed to use a condom with client as a result of own drinking during the past 12 months. The heavy black lines indicate the cumulative RDS-II weighted estimate overall for each site, while the grey lines are unweighted proportions for each seed, by sample wave.

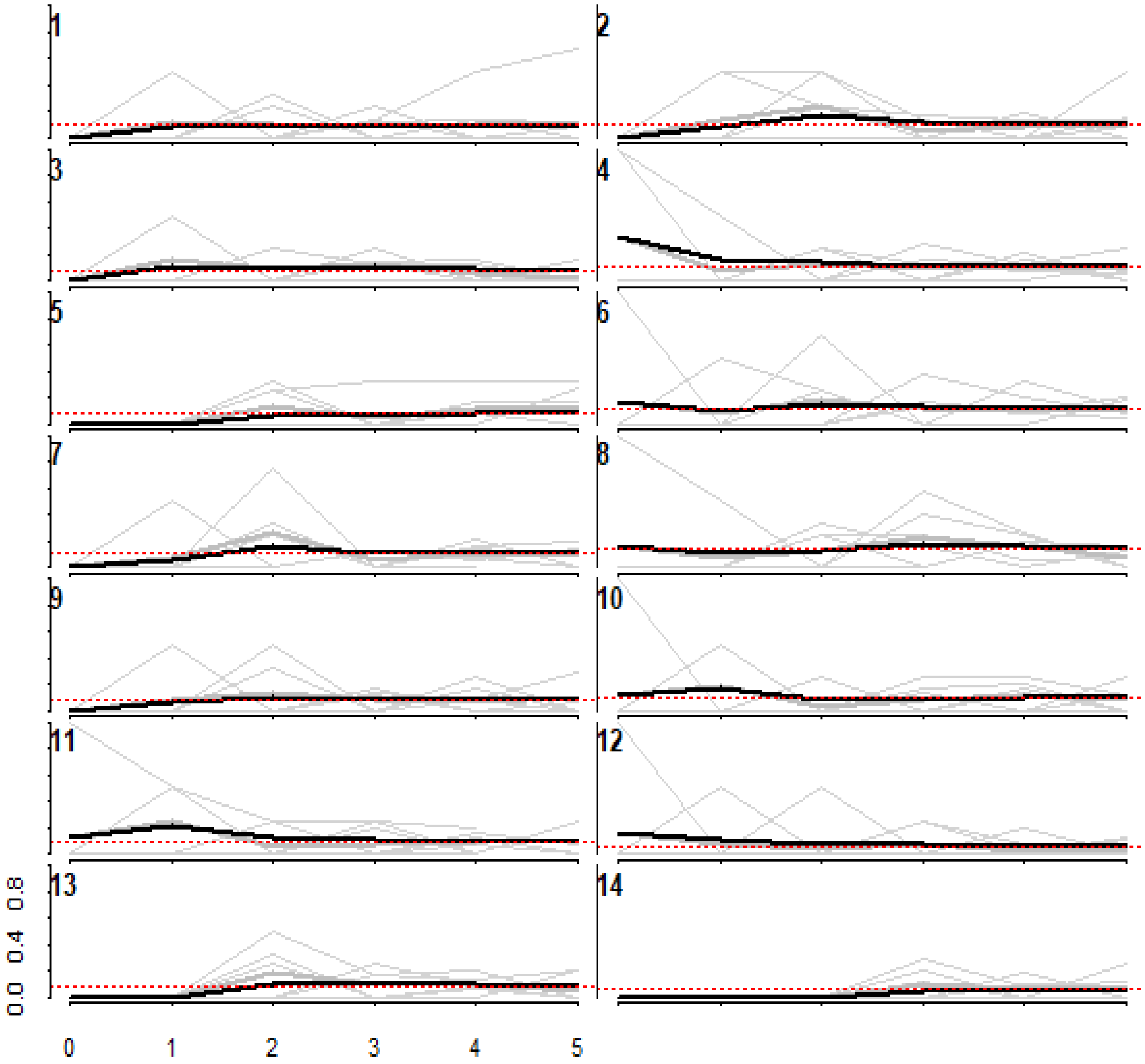

Figure 24: Convergence of the proportion of FSW who reported to have failed to use a condom with a client as a result of client's drinking during the past 12 months. The heavy black lines indicate the cumulative RDS-II weighted estimate overall for each site, while the grey lines are unweighted proportions for each seed, by sample wave.

Table 1: Recruitment homophily for prevalent HIV

Site	2013 survey		2016 survey	
	Homophily for age	Homophily for prevalent HIV	Homophily for age	Homophily for prevalent HIV
1	1.0	1.1	1.0	1.0
2	1.0	1.0	1.1	1.0
3	1.1	1.2	1.0	1.1
4	1.1	1.2	1.1	1.0
5	1.0	1.1	1.0	1.0
6	1.0	1.1	1.0	1.0
7	1.0	1.1	1.0	1.2
8	1.1	1.1	1.0	0.9
9	1.1	1.1	1.0	1.1
10	1.0	1.1	1.1	1.0
11	1.0	1.2	1.0	1.1
12	1.2	1.0	1.1	1.0
13	1.1	1.0	1.1	1.1
14	1.0	1.0	1.0	1.0

Appendix 2: Table of factors associated with prevalent HIV in 2013

Table 2: Factors associated with prevalent HIV among young FSW in 2013 (N=656

Characteristic		N (%)	Number of young female sex-workers tested HIV-positive during the survey (n=236) n (%)	Crude OR (95%CI)	P-value	Age adjusted OR (95%CI)	P-value	Adjusted* OR (95%CI)	P-value*
Age at time of survey					0.067				0.125
	18-19	108 (17.4)	28 (26.7)	1				1	
	20-24	547 (82.6)	208 (37.0)	1.94 (0.95-3.96)				1.75 (0.86-3.58)	
Marital status					0.040		0.106		0.127
	Never married	246 (37.3)	69 (26.8)	1		1		1	
	Ever married	409 (62.7)	167 (40.2)	1.68 (1.02-2.75)		1.72 (0.83-3.54)		1.48 (0.89-2.44)	
Highest level of education					0.479				
	Primary school or less	152 (25.2)	60 (41.3)	1					
	Some secondary school	281 (45.3)	108 (36.5)	0.97 (0.54-1.76)					
	Complete secondary or higher	220 (29.5)	68 (28.3)	0.72 (0.38-1.37)					
Number of children									
	0	197 (33.1)	74 (37.2)	1	0.515				
	1-2	381 (57.4)	136 (34.7)	0.80 (0.47-1.36)					
	≥3	77 (9.5)	26 (31.3)	0.65 (0.29-1.43)					
Duration in sex work					0.351				
	0-2	381 (63.8)	121 (32.4)	1					
	3-4	187 (24.2)	83 (43.1)	1.46 (0.86-2.51)					
	≥5	87 (12.0)	32 (34.5)	0.99 (0.47-2.11)					
Number of clients in the last week					0.165				
	0-4	271 (47.9)	94 (32.6)	1					
	5-9	165 (25.2)	51 (29.2)	0.72 (0.40-1.27)					
	≥10	219 (26.9)	91 (45.6)	1.32 (0.76-2.29)					
Condom use at last sex with steady partner					0.369				
	No	183 (46.4)	69 (36.8)	1					
	Yes	211 (53.6)	62 (28.9)	0.75 (0.41-1.40)					
Condom-less sex with steady partner in the past month					0.653				
	No	145 (67.8)	41 (27.2)	1					
	Yes	66 (32.2)	21 (32.4)	1.22 (0.51-2.91)					

Condom use at last sex with client									
	No	112 (15.3)	38 (38.2)	1	0.852				
	Yes	543 (84.7)	198 (34.7)	0.93 (0.44-1.98)					
Condom-less sex with client in the past month					0.447				
	No	351 (59.9)	118 (31.8)	1					
	Yes	247 (40.1)	92 (38.9)	1.21 (0.74-1.98)					
Experience of physical violence from steady partner					0.762				
	No	368 (60.3)	120 (32.1)	1					
	Yes	287 (39.7)	116 (40.0)	1.08 (0.67-1.73)					
Experience of physical violence from client					0.424				
	No	478 (76.3)	172 (34.9)	1					
	Yes	177 (23.7)	64 (36.1)	0.80 (0.46-1.38)					
Ever forced to have sexual intercourse					0.265				
	No	627 (95.3)	225 (34.4)	1					
	Yes	28 (4.7)	11 (51.8)	1.62 (0.69-3.78)					
Alcohol use in the past 12 months					0.464				
	Never	226 (38.2)	82 (39.9)	1					
	Once a month or less	67 (11.4)	22 (22.8)	0.49 (0.23-1.06)					
	2-4 times per month	91 (12.9)	33 (34.9)	0.91 (0.45-1.81)					
	2-3 times per week	101 (14.2)	37 (36.3)	0.90 (0.43-1.89)					
	4 or more times per week	170 (23.3)	62 (33.1)	0.76 (0.40-1.43)					
No food for one day in the past month					0.680				
	No	394 (60.4)	139 (35.6)	1					
	Yes	261 (39.6)	97 (34.6)	0.90 (0.56-1.47)					
Relationship with other FSW in one's location					0.236				
	Good	385 (61.0)	148 (38.8)	1					
	Neither good nor bad	205 (28.8)	63 (28.7)	0.65 (0.38-1.10)					
	Bad or no relationship	65 (10.2)	25 (31.9)	0.68 (0.31-1.51)					
No. of FSW who are close friends					0.096		0.090		0.101
	≤1	186 (25.6)	81 (43.6)	1		1		1	
	2-3	348 (56.8)	117 (33.4)	0.71 (0.40-1.25)		0.72 (0.41-1.28)		0.73 (0.42-1.30)	
	≥4	121 (17.6)	38 (28.8)	0.47 (0.23-0.93)		0.46 (0.23-0.92)		0.47 (0.23-0.94)	
Symptoms of common mental disorder					0.342				
	No	326 (51.9)	100 (30.4)	1					
	Yes	326 (48.1)	135 (40.6)	1.26 (0.78-2.02)					

*Adjusted for age at survey, marital status and number of FSW who are close friends

References

1. Cowan FM, Davey CB, Fearon E, Mushati P, Dirawo J, Cambiano V, et al. The HIV Care Cascade Among Female Sex Workers in Zimbabwe: Results of a Population-Based Survey From the Sisters Antiretroviral Therapy Programme for Prevention of HIV, an Integrated Response (SAPPH-IRe) Trial. Journal of acquired immune deficiency syndromes (1999). 2017;74(4):375-82.

2. Cowan FM, Davey C, Fearon E, Mushati P, Dirawo J, Chabata ST, et al. Targeted combination prevention to support female sex workers in Zimbabwe accessing and adhering to antiretrovirals for treatment and prevention of HIV (SAPPH-IRe): a cluster-randomised trial. The lancet HIV. 2018;5(8):e417-e26.
